# Metabolites augment oxidative stress to sensitize antibiotic-tolerant *Staphylococcus aureus* to fluoroquinolones

**DOI:** 10.1128/mbio.02714-24

**Published:** 2024-10-30

**Authors:** Jonathan I. Batchelder, Andrew J. Taylor, Wendy W. K. Mok

**Affiliations:** 1 Department of Molecular Biology and Biophysics, UConn Health, Farmington, Connecticut, USA; 2 Department of Biomedical Engineering, Boston University, Boston, Massachusetts, USA; The Hebrew University of Jerusalem, Jerusalem, Israel

**Keywords:** fluoroquinolones, antibiotic persistence, oxidative stress, antibiotic stress response, metabolism, *Staphylococcus aureus*

## Abstract

If left unchecked, infections involving antibiotic-refractory bacteria are expected to cause millions of deaths per year in the coming decades. Beyond genetically resistant bacteria, persisters, which are genetically susceptible cells that survive antibiotic doses that kill the rest of the clonal population, can potentially contribute to treatment failure and infection relapse. Stationary-phase bacterial cultures are enriched with persisters, and it has been shown that stimulating these populations with exogenous nutrients can reduce persistence to different classes of antibiotics, including topoisomerase-targeting fluoroquinolones (FQs). In this study, we show that adding glucose and amino acids to nutrient-starved *Staphylococcus aureus* cultures enhanced their sensitivity to FQs, including delafloxacin (Dela)—a drug that was recently approved for treating staphylococcal infections. We found that while the added nutrients increased nucleic acid synthesis, this increase was not required to sensitize *S. aureus* to FQs. We further demonstrate that addition of these nutrients increases membrane potential and the ability to generate harmful reactive oxygen species (ROS) during FQ treatment. Chelating iron, scavenging hydroxyl radicals, and limiting oxygenation during FQ treatment and during recovery following FQ treatment rescued nutrient-stimulated *S. aureus*. In all, our data suggest that while nutrient stimulation increases the activity of FQ targets in stationary-phase *S. aureus*, the resulting generation of ROS, presumably made possible through metabolic upregulation, is the primary driver of increased sensitivity to these drugs.

**IMPORTANCE:**

*Staphylococcus aureus* causes many chronic and relapsing infections because of its ability to endure host immunity and antibiotic therapy. While several studies have focused on the nutrient requirements for the formation and maintenance of staphylococcal infections, the effects of the nutrient environment on bacterial responses to antibiotic treatment remain understudied. Here, we show that adding nutrients to starved *S. aureus* activates biosynthetic processes, including DNA synthesis, but it is the generation of harmful reactive oxidants that sensitizes *S. aureus* to DNA topoisomerase-targeting FQs. Our results suggest that the development of approaches aimed at perturbing metabolism and increasing oxidative stress can potentiate the bactericidal activity of FQs against antibiotic-tolerant *S. aureus*.

## INTRODUCTION

Antibiotic treatment failure complicates efforts to cure bacterial infections, leading to chronic infections, increased morbidity, and in many cases, mortality (1–4). With the number of deaths caused by antibiotic-resistant bacteria expected to increase in the coming years, it is essential that we improve our current approaches to preserve the efficacy of our existing antibiotics and ensure that we can continue to eradicate infections (5, 6). Among the pathogens of serious concern in our healthcare system, drug-resistant *Staphylococcus aureus* is especially burdensome—it was associated with the deaths of 750,000 individuals worldwide in 2019 (7).

Aside from drug-resistant bacteria, antibiotic-susceptible cultures can harbor small subpopulations of phenotypically tolerant cells, called persisters, that survive antibiotics administrated at doses that kill their clonal kin (8). Persisters are thought to contribute to chronic infections and relapses, and recent studies of many bacterial species suggest that persister progenies may have an increased likelihood of developing heritable antibiotic resistance (1, 9–16). Therefore, the survival of persisters may serve as a gateway not only to infection recurrence but also to the development and dissemination of resistance, underscoring the need to limit antibiotic-refractory persisters.

It is well established that biosynthetic and metabolic activities modulate bacterial persistence to antibiotic treatment (17–21). Bacteria in nutrient-limited stationary-phase cultures are far more likely to persist than actively growing bacteria (9, 22–25). Our group and others have previously reported that fluoroquinolone (FQ) antibiotics, which target DNA topoisomerases, retain bactericidal activity against non-growing cultures of *S. aureus* and other bacterial species (26–29). Although the ability of FQs to kill stationary-phase bacteria is markedly reduced compared to exponentially growing cells, these drugs can still eradicate >90% of many stationary-phase cultures (25–27, 30). Understanding how the nutrient environment impacts *S. aureus* persistence to FQs is relevant because FQs are used clinically to treat *S. aureus* infections, many of which occur in nutrient-depleted host sites (31–36). For example, the FQs delafloxacin (Dela), moxifloxacin (Moxi), and ciprofloxacin (Cipro) are all FDA-approved for treating *S. aureus* skin and skin structure infections (37–39). These infections are characterized by abscesses, which are limited in glucose, the preferred carbon source of *S. aureus* (40). Additionally, Moxi and Dela are approved to treat staphylococcal respiratory infections, where *S. aureus* has been reported to undergo extensive transcriptional remodeling to adapt to nutrient-limited airways (37, 38, 41). Beyond FDA-approved usage, Moxi and Cipro have been used to treat chronic staphylococcal osteomyelitis, which is often limited for purines, aspartate, or aspartate-derived amino acids (42, 43).

Building on these observations and the need to improve the efficacy of these drugs against *S. aureus*, several labs have shown that adding nutrients to stationary-phase cultures of bacteria, including *S. aureus*, can enhance killing by FQs and other antibiotics (28, 44, 45). In some cases, nutrient stimulation increases drug uptake and/or the activity of enzymes that the antibiotics target, thereby sensitizing stationary-phase bacteria to the drugs (26, 44). Consistent with this postulate, results from our lab show that stimulating stationary-phase *Escherichia coli* with glucose increases transcription and topoisomerase activity, resulting in decreased persistence to the FQ levofloxacin (26). However, it is not known whether activating transcription is important for sensitizing stationary-phase *S. aureus* to FQs. To date, the few studies on potentiating FQs against *S. aureus* persisters have focused on older FQ compounds, such as Cipro, that are not often used to treat *S. aureus* infections due to increasing resistance to these drugs (28, 46, 47). Additionally, the previous study on nutrient sensitization of *S. aureus* to FQs focused on adding glucose, leaving the effects of other important nutrients, such as amino acids and nucleobases, unknown (28).

Given these knowledge gaps, we set out to determine the effects of added nutrient sources on the persistence of stationary-phase *S. aureus* to the newer clinically relevant FQs, Dela and Moxi, and found that a combination of glucose and amino acids sensitized these cells to FQs. Our data suggest that adding nutrients stimulated nucleic acid synthesis in stationary-phase *S. aureus*, but this is not required to potentiate the bactericidal activity of FQs. We further show that the metabolites increased flux through the electron transport chain, which enhanced reactive oxygen species (ROS) generation and bolstered the efficacy of FQs against stationary-phase *S. aureus*.

## RESULTS

### Stimulating stationary-phase *S. aureus* with glucose + amino acids decreases FQ persistence

To assess the impact of added nutrients on stationary-phase *S. aureus* persistence, we cultured *S. aureus* strain 43300, a methicillin-resistant strain, for 17 h in a rich defined medium (RDM). We confirmed that at this point, the population was in stationary phase (Fig. S1A) and was not susceptible to vancomycin (Vanco), which is expected for a slow-/non-growing culture that is not undergoing extensive cell wall synthesis (Fig. S1B). We treated these cells with a range of Dela, Moxi, and Cipro doses (Fig. S1C through E). Populations treated with Dela and Moxi exhibited biphasic concentration-dependent survival curves, but essentially no killing following Cipro treatment was detected, consistent with reports showing that the newer FQs are more effective against *S. aureus* than Cipro (48, 49). We sought to determine the nutrients in RDM that can potentiate FQs against stationary-phase *S. aureus* and elucidate the cellular processes that need to be reactivated for sensitization to these drugs. We focused primarily on Dela since this drug was recently approved, and, to our knowledge, no research has been done on improving its efficacy against *S. aureus*.

**Fig 1:**
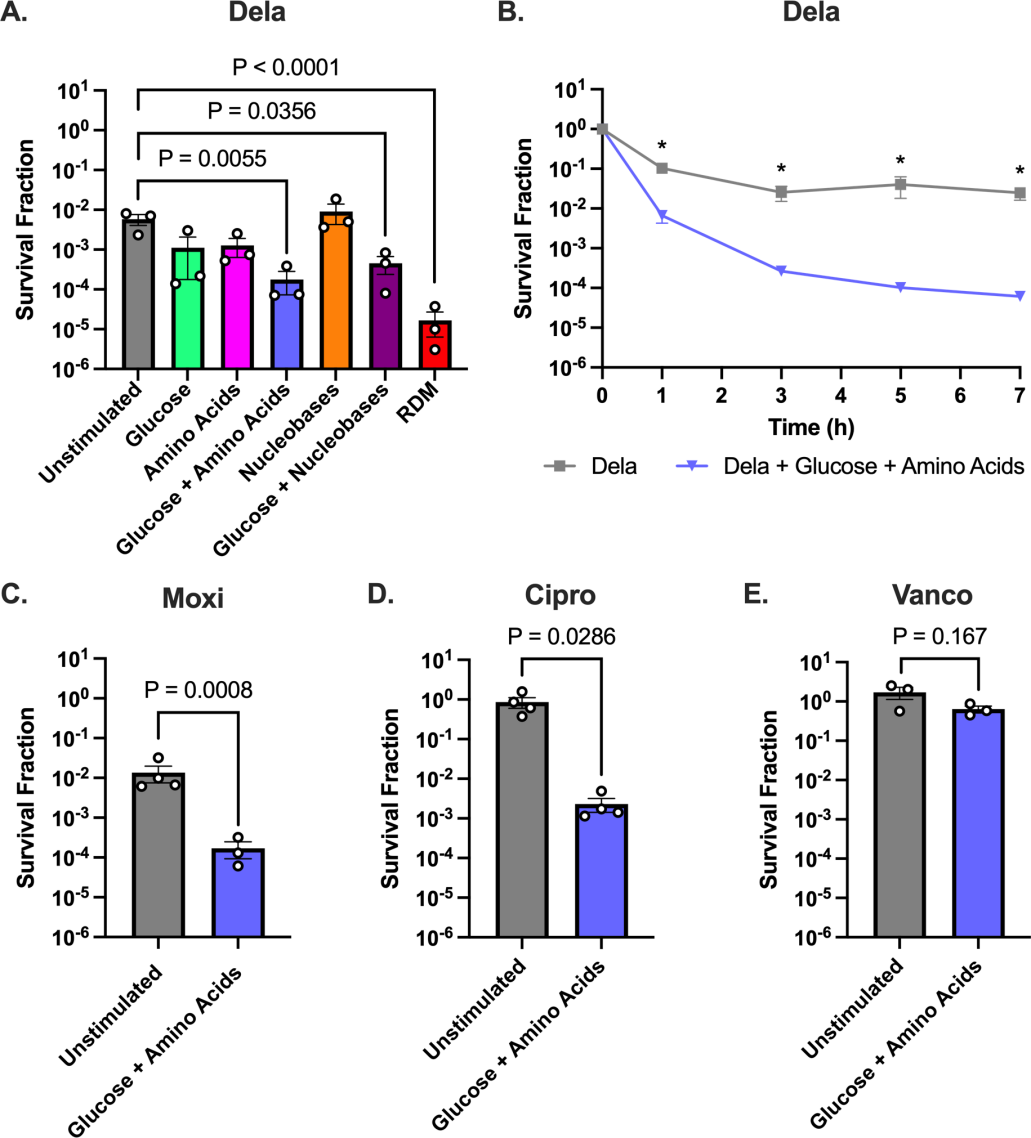
Glucose + amino acids decrease persistence of stationary-phase *S. aureus* to FQs. (**A**) Metabolite screen comparing survival of *S. aureus* 43300 after 7 h of Dela treatment in the presence of various nutrients. (**B**) Time-dependent kill curve showing survival of 43300 throughout 7 h of Dela treatment in the presence vs absence of glucose + amino acids. (**C–E**) Survival of *S. aureus* 43300 after 7 h of treatment with (**C**) Moxi, (**D**) Cipro, or (**E**) Vanco in the presence or absence of glucose +amino acids. At least three independent replicates were performed for each experiment. *P* values were calculated by comparing the log-transformed values of each condition to the unstimulated condition treated with Dela (**A**) using Dunnett’s multiple comparisons test following analysis of variance (ANOVA) or (**B–E**) two-tailed *t*-tests. *P < 0.05. Error bars denote SEM.

RDM contains three major groups of nutrients as follows: glucose, amino acids, and nucleobases. Therefore, we decided to test whether each of these groups alone or in combination could sensitize stationary-phase *S. aureus* to Dela. As a positive control, we replenished all three groups of nutrients by adding RDM before treating stationary-phase *S. aureus* with 5 µg/mL of Dela (2,500× minimum inhibitory concentration [MIC]) and observed significantly decreased survival (Fig. 1A). By comparison, the addition of nucleobases alone did not reduce *S. aureus* survival. Adding glucose or amino acids alone decreased survival fivefold (not statistically significant), but the combination of glucose + amino acids or glucose + nucleobases resulted in a significant decrease in survival. Since glucose + amino acids had the largest effect, second only to adding complete RDM, we chose to focus on these nutrients.

When we treated stationary-phase *S. aureus* with Dela with or without added glucose + amino acids over a 7-h period, we detected biphasic killing, indicating that the surviving cells after 7 h of treatment were persisters and that the added metabolites decreased persistence to Dela (Fig. 1B). Therefore, we treated *S. aureus* with antibiotics for 7 h in the rest of the survival experiments. We found that stimulating *S. aureus* with glucose + amino acids increased cell death by ~100-fold following treatment with other FQs, including Moxi (10 µg/mL; 500× MIC) and Cipro (50 µg/mL; 250× MIC), indicating that the effect of nutrient stimulation was not exclusive to Dela (Fig. 1C and D). However, these nutrients did not sensitize *S. aureus* to Vanco, indicating that at least for the drugs we tested, this effect was FQ-specific (Fig. 1E).

### Metabolic stimulation increases DNA and protein synthesis

Consistent with previous studies, which focused mainly on *E. coli*, we show that stimulating high-density, stationary-phase *S. aureus* cultures with added metabolites sensitized the population to FQs (26, 28). We then asked how the added metabolites sensitize non-growing *S. aureus* to these topoisomerase inhibitors. The observed increase in FQ lethality upon stimulating *S. aureus* with glucose + amino acids is reminiscent of stringent response reversal upon replenishment of nutrients following starvation, which leads to increased intracellular GTP levels and the resumption of many biosynthetic activities (50, 51). We first tested whether these nutrients trigger growth resumption of stationary-phase *S. aureus*. We found no significant increases in OD_600_ or CFU/mL during the first hour after stimulation (Fig. S2), suggesting that cell division had not resumed by the time the cells were treated with FQs. We then sought to determine whether the biosynthetic processes targeted by FQs were stimulated by these nutrients even if proliferation had not yet resumed.

FQs inhibit DNA topoisomerases, which relieve supercoiling in the DNA double helix during RNA and DNA synthesis (52–57). We hypothesized that adding glucose + amino acids stimulated RNA or DNA synthesis, which increased the number of active topoisomerases the FQs could inhibit. To test this hypothesis, we measured the incorporation of radioactive ^3^H-uridine into newly synthesized nucleic acids after exposure to these nutrients. Additionally, since translation and transcription are coupled in bacteria, we measured protein synthesis as well (58).

**Fig 2:**
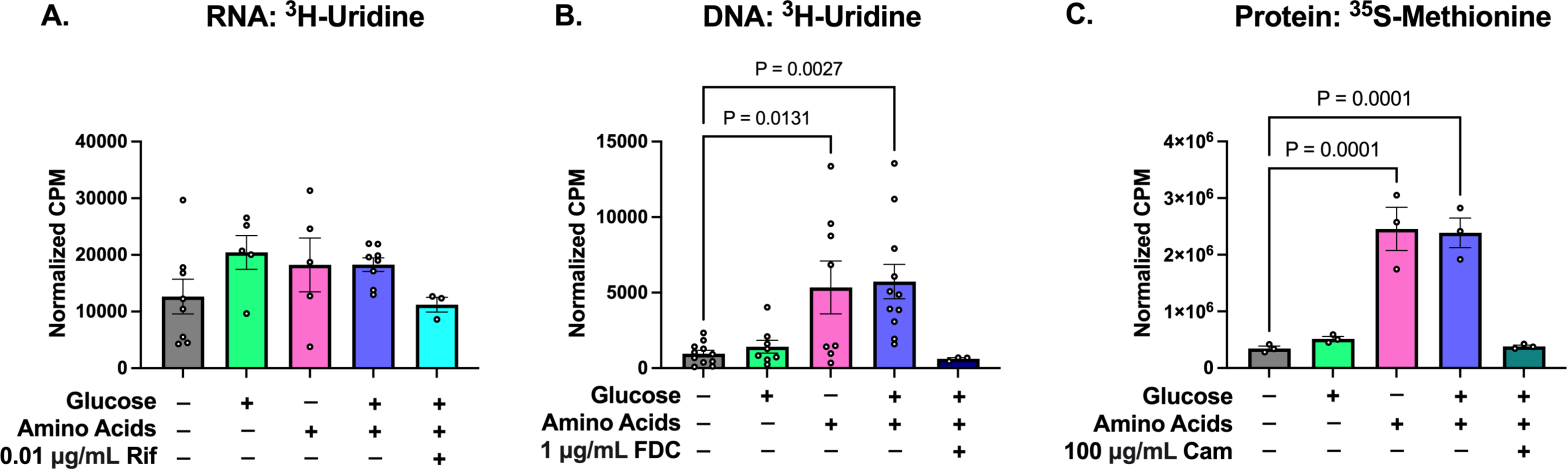
Impact of nutrient stimulation on macromolecular biosynthesis. (**A**) RNA synthesis and (**B**) DNA synthesis were measured using the incorporation of ^3^H-uridine. (**C**) Protein synthesis was measured using the incorporation of ^35^S-methionine. Radiolabeled nucleic acids and proteins were detected by scintillation counting and reported as counts per minute (CPM) normalized by the OD_600_ of each sample. At least three independent replicates were performed for each experiment. *P* values were calculated comparing each condition to the unstimulated condition using Dunnett’s multiple comparisons test following ANOVA. Error bars denote SEM.

The levels of RNA, DNA, and protein synthesis were all significantly lower in stationary-phase than in exponentially growing *S. aureus*, as expected (Fig. S3). However, transcriptional activity remained high even in the absence of metabolic stimulation (Fig. 2A), presumably using recycled ribonucleotides from transcript turnover (59). Glucose, amino acids, and glucose + amino acids all increased transcription levels, but none of these changes was statistically significant. Contrary to RNA synthesis, we found that stationary-phase *S. aureus* did not have high DNA replication activity and that adding glucose did not appreciably increase DNA synthesis (Fig. 2B). By comparison, adding amino acids or glucose + amino acids significantly increased DNA synthesis. Finally, we found that adding amino acids alone or glucose + amino acids significantly increased protein synthesis (Fig. 2C).

### Increased nucleic acid synthesis is not required for nutrient sensitization to FQs

Given that glucose + amino acids increased transcription, replication, and translation levels compared to unstimulated cells, we next asked whether increased levels of these processes were required for the observed sensitization to FQs. We reasoned that if an increase in a given biosynthetic process was required for enhanced FQ lethality, then inhibiting that process to unstimulated levels in stimulated *S. aureus* would protect the cells. We used 0.01 µg/mL rifampicin (Rif; 1.25× MIC), 1 µg/mL 5-fluoro-2′-deoxycytidine (FDC; 1,000× MIC), and 100 µg/mL chloramphenicol (Cam; 10× MIC) to inhibit transcription, DNA replication, and translation, respectively. These doses were chosen because they inhibited RNA, DNA, and protein synthesis in glucose + amino acids-stimulated cells to levels that were comparable to unstimulated cells (Fig. 2A through C). Rather than completely shutting off these processes, our goal was to determine whether increased biosynthetic activity beyond the levels detected in unstimulated cells was necessary for sensitization to FQs. To ensure that these processes were being inhibited at the time of FQ treatment, we pre-treated the stimulated cells for 30 min with a given inhibitor then continued treating with this inhibitor throughout the subsequent FQ treatment.

**Fig 3:**
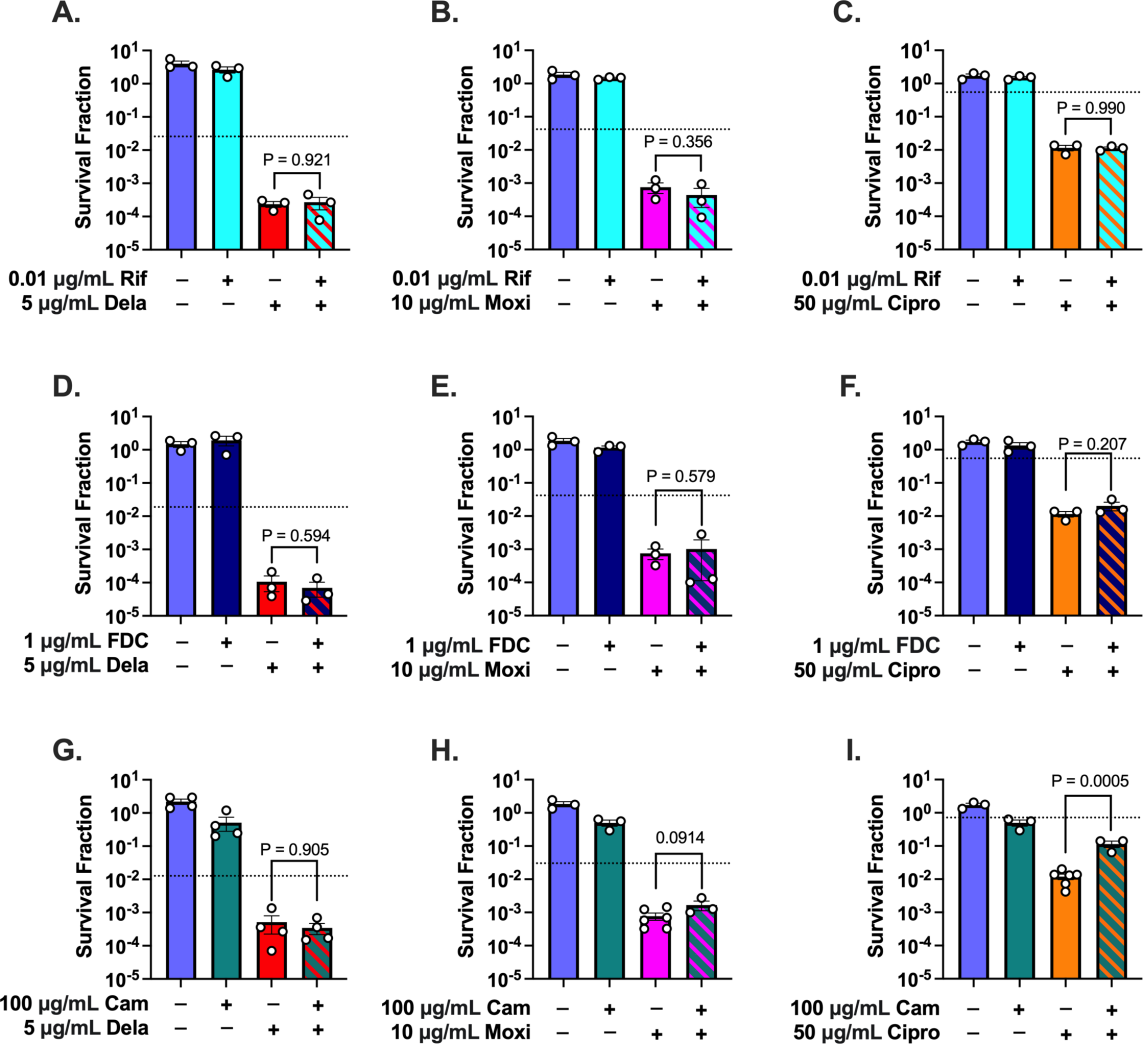
Inhibition of nucleic acid synthesis to levels detected in unstimulated cultures fails to reverse nutrient sensitization to FQs. Cells were pre-treated for 30 min with (**A–C**) Rif, (**D–F**) FDC, or (**G–I**) Cam during exposure to glucose + amino acids and then throughout 7 h of treatment with Dela, Moxi, or Cipro. Dotted lines indicate the survival of unstimulated FQ-treated cells in a given experiment. At least three biological replicates were performed for each experiment. *P*-values were calculated comparing the log-transformed values of glucose + amino acids + FQ vs glucose + amino acids + FQ + inhibitor using two-tailed *t*-tests. Error bars denote SEM.

We found that inhibiting transcription in metabolically stimulated *S. aureus* with Rif before and during treatment with Dela, Moxi, or Cipro failed to rescue the cells from the increased killing caused by the nutrients (Fig. 3A through C). Since a single-nucleotide mutation in *rpoB* is sufficient to confer Rif resistance, we ensured that the lack of rescue was not due to the presence of Rif-resistant mutants that take over the culture (Fig. S4) (60). Additionally, we also treated the stimulated cultures with 10-fold more Rif (0.1 µg/mL). This dose inhibited transcription in stimulated cells to 1/10 that of unstimulated cells and killed ~90% of the stimulated population even without FQ (Fig. S5A and B). This dose also failed to increase the survival of Dela-treated cells. These results are consistent with a previous report showing that Rif failed to rescue Cipro-treated exponential-phase *S. aureus* (24). Additionally, these data demonstrate that the requirements for nutrient sensitization of *S. aureus* to FQs are distinct from those for *E. coli*, as transcription inhibition protected stationary-phase *E. coli* from FQs following metabolic stimulation (26).

Similar to what we found when inhibiting transcription, inhibiting DNA synthesis to levels comparable to unstimulated cells failed to rescue the stimulated cells from FQs (Fig. 3D through F). We also found that decreasing translation to unstimulated levels failed to rescue stimulated cells from Dela or Moxi (Fig. 3G and H), although Cam did cause a sixfold increase in the survival of stimulated cells treated with Cipro (Fig. 3I). Collectively, our data suggest that increasing *de novo* nucleic acid synthesis is not sufficient to explain why nutrient stimulation sensitizes *S. aureus* to FQs. These data imply that perturbations beyond increasing primary target activity are responsible for the increased killing.

### Stimulating cells with glucose + amino acids increases adenylate charge and membrane potential

**Fig 4:**
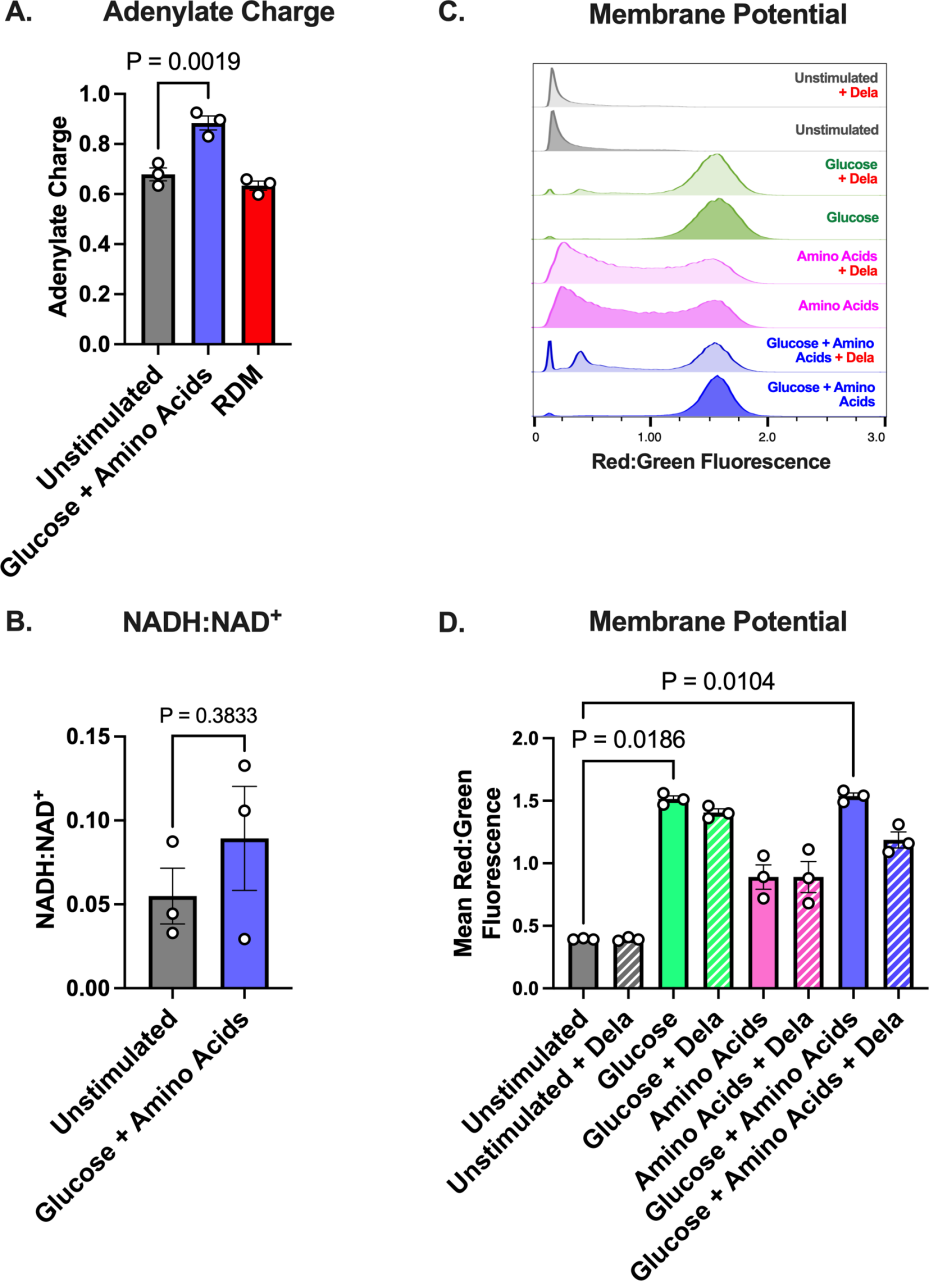
Metabolic activity of stationary-phase *S. aureus* 43300 in the presence of nutrient stimuli. Stationary-phase *S. aureus* cultures were given various nutrients for 1 h before their (**A**) adenylate charge, (**B**) NADH:NAD^+^ ratio, and (**C, D**) membrane potential were quantified. (**A**) Adenylate charge, a measure of the energy stored in the adenylate nucleotide pool, was calculated using the following formula: ([ATP] + 0.5[ADP])/([AMP] + [ADP] + [ATP]). (**C, D**) Flow cytometry histograms of red:green fluorescence ratio are shown in (**C**), whereas (**D**) shows the quantification of mean red:green fluorescence ratio. At least three biological replicates were performed for each experiment, and the flow cytometry histograms shown are representative of these replicates. For panel B, t-test was used rather than Dunnett's and ANOVA since there are only two conditions being compared. *P* values were calculated comparing each experimental condition to the unstimulated condition using Dunnett’s multiple comparisons test following ANOVA. Error bars denote SEM.

Given that changes in the levels of RNA, DNA, and protein synthesis failed to explain the increase in FQ lethality against *S. aureus* stimulated with glucose + amino acids, we asked whether adding these nutrients reenergized the cells. Several facets of bacterial metabolism, including ATP levels, concentrations of electron carriers, and electron transport chain (ETC) activity have previously been linked to the ability of bactericidal antibiotics to kill cells (24, 61, 62). Interestingly, we found that glucose + amino acids-stimulated cells had a significantly higher adenylate charge than unstimulated cells, whereas cells given complete RDM had adenylate charges comparable to unstimulated populations (Fig. 4A). The total adenylate nucleotide pool increased in cells given RDM, but it remained unchanged in glucose + amino acids-treated cells (Fig. S6). These data are consistent with a previous report that showed decreased persistence in *S. aureus* cells with increased ATP or energy charge (24).

The ratio of NADH (an important electron carrier) to NAD^+^ increased ~60% (Fig. 4B). While this is not statistically significant, it is nonetheless consistent with altered redox balance and could affect ETC activity. Given that Lobritz and colleagues showed that decreases in cellular respiration protect bacteria, including *S. aureus*, from bactericidal antibiotics, we hypothesized that nutrient stimulation was increasing ETC activity, leading to increased lethality (62).

As a proxy for measuring ETC activity, we measured membrane potential (ΔΨ) using the potential-sensitive dye DiOC_2_(3) in the presence and absence of Dela. DiOC_2_(3) accumulates more within cells that have a greater proton gradient and self-quenches, leading to a shift toward increased red fluorescence when ΔΨ is increased (63). As a control experiment, we showed that the protonophore carbonyl cyanide *m*-chlorophenyl hydrazone (CCCP) depolarizes exponential-phase cells as indicated by low red:green fluorescence (Fig. S7A and B). As expected, we found that unstimulated cells had relatively low ΔΨ (Fig. 4C and D; Fig. S7C). Cells stimulated with amino acids had increased ΔΨ compared to unstimulated cells, and the glucose and glucose + amino acids conditions had the greatest ΔΨ.

In populations stimulated with glucose or glucose + amino acids, a subpopulation of cells shifted toward lower red:green fluorescence during Dela treatment (Fig. 4C and D; Fig. S7C). We posit that the peaks with lower red:green fluorescence may represent dead or dying cells whose proton gradients have dissipated. However, the majority of cells in these populations exhibited higher red:green fluorescence, which indicates that these cells maintained high ΔΨ at this point during treatment. These data suggest that glucose + amino acids stimulate ETC activity, consistent with increased metabolic activity that may contribute to increased antibiotic lethality.

### Glucose + amino acids lead to increased levels of reactive oxygen species during FQ treatment

Kohanski and colleagues previously proposed that once bactericidal antibiotics damage their primary targets, the cell reprograms metabolism as it attempts to repair this damage (64). These perturbations, particularly an increase in ETC activity, ultimately result in the generation of harmful ROS, which contribute to cell death (16, 64–68). We hypothesized that under stimulated conditions, FQ-treated *S. aureus* would have higher ROS levels because they have more nutrients available to drive central energy metabolism upon incurring damage.

To determine how nutrient stimulation and FQ treatment affect ROS levels in stationary-phase *S. aureus*, we used the carboxy-H_2_DCFDA assay. Carboxy-H_2_DCFDA crosses bacterial membranes as a non-fluorescent dye, is cleaved by esterases, then reacts with several types of ROS, including hydroxyl and peroxyl radicals, to yield a fluorescent product (69, 70). Increased fluorescence intensity correlates with elevated ROS levels, making the assay useful as a qualitative gauge of relative ROS levels between populations, but not as a quantitative measure of absolute ROS concentrations. We confirmed that this assay works as expected in *S. aureus* by treating cells stimulated with glucose + amino acids with tert-butyl hydroperoxide (TBHP, a known inducer of oxidative stress), thiourea (TU, an antioxidant), or TBHP + TU (27, 64, 71, 72). We demonstrated that TBHP led to a significantly larger percentage of cells having higher ROS levels than populations given only glucose + amino acids or treated with both TBHP and TU (Fig. S8A and B).

**Fig 5:**
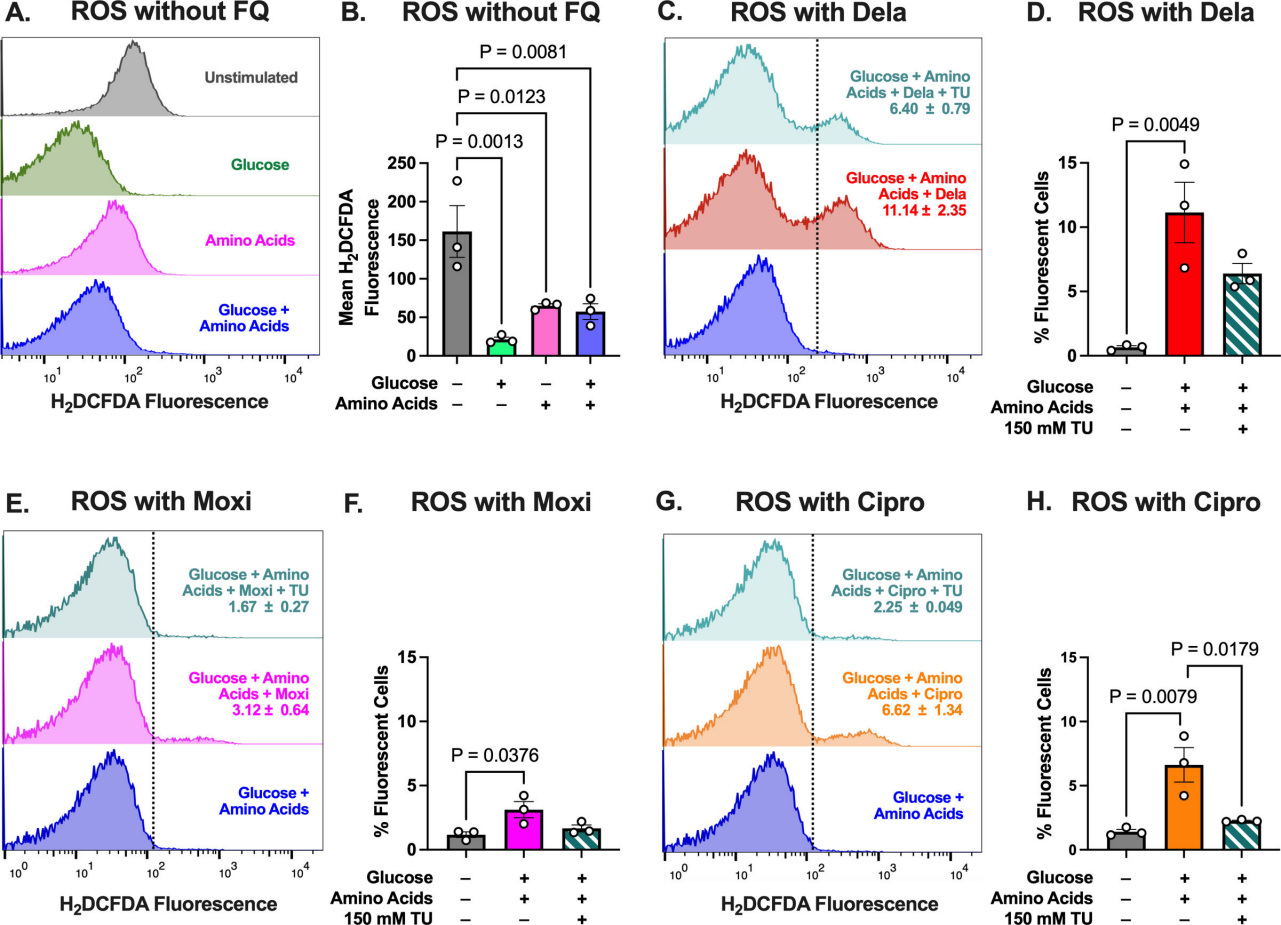
Nutrient stimulation increases oxidative stress during FQ treatment. (**A**) Flow cytometry histograms of H_2_DCFDA fluorescence in unstimulated cells and cells given various nutrients. (**B**) Quantification of mean of H_2_DCFDA fluorescence shown in (**A**). (**C, E, G**) Flow cytometry histograms of H_2_DCFDA fluorescence in cells given glucose + amino acids with (**C**) Dela, (**E**) Moxi, or (**G**) Cipro. A total of 99% of cells that were not treated with a given FQ lie to the left of the dashed lines. Histograms are representative of three independent replicates. The values in the histogram denote the mean percentage of cells that, during treatment with a given FQ, had higher fluorescence than non-FQ-treated cells ± SEM of three independent replicates. These values are quantified in panels (D), (F), and (H), respectively. *P*-values were calculated comparing (**B**) each condition to the unstimulated condition using Dunnett’s multiple comparisons test following ANOVA or (**D, F, H**) each condition to every other condition using Dunnett’s multiple comparisons test following ANOVA. Error bars denote SEM.

In the absence of FQ, unstimulated cells had higher ROS levels than cells stimulated with any of the nutrient groups (Fig. 5A and B; Fig. S8C), consistent with observations showing increased oxidative stress in stationary-phase *S. aureus* (73, 74). We hypothesize that the addition of nutrients to these cells allows them to engage oxidative stress responses, perhaps through higher expression of ROS-detoxifying genes.

Given that the cells in different nutrient conditions had varying baseline ROS levels without FQ treatment, we determined the percentage of cells that exhibited increased ROS after 1 h of FQ treatment under each condition. Strikingly, none of the FQs further increased ROS in unstimulated cells (Fig. S8D through F). Dela treatment did not appreciably increase ROS in cells stimulated with amino acids (Fig. S8G). By comparison, Dela treatment led to increased ROS levels in ~8% of cells stimulated with glucose (Fig. S8H), and all three FQs increased ROS levels in cells stimulated with glucose + amino acids (Fig. 5C through H; Fig. S8I through L). Interestingly, Dela increased ROS the most in *S. aureus*, while Moxi had the least effect, implying that while these nutrients potentiate all three FQs to approximately the same extent (decreasing survival about 100-fold), the oxidative stress levels induced by the different FQs vary. We further showed that TU partially reduced ROS levels in FQ-treated cells stimulated with glucose + amino acids. These results support our hypothesis that the added glucose + amino acids enable metabolic processes that contribute to ROS generation upon FQ treatment in *S. aureus*.

### Reducing oxidative stress rescues nutrient-stimulated *S. aureus* from increased FQ lethality

Given our results showing increased ROS upon FQ treatment in nutrient-stimulated *S. aureus*, we next asked whether this increased oxidative stress is responsible for the enhanced FQ bactericidal activity. To answer this question, we exposed unstimulated and glucose + amino acids-stimulated *S. aureus* to two antioxidants, 2,2′-bipyridine (Bipy) and thiourea (TU) throughout FQ treatment. Bipy chelates Fe^2+^ and thereby prevents it from reacting with H_2_O_2_ to produce harmful radicals via the Fenton cycle (64, 75). TU is suggested to scavenge hydrogen peroxide as well as hydroxyl and superoxide radicals (72). Adding these compounds during FQ treatment is expected to decrease oxidative stress in *S. aureus* and limit damage to cellular macromolecules that could contribute to increased lethality.

**Fig 6:**
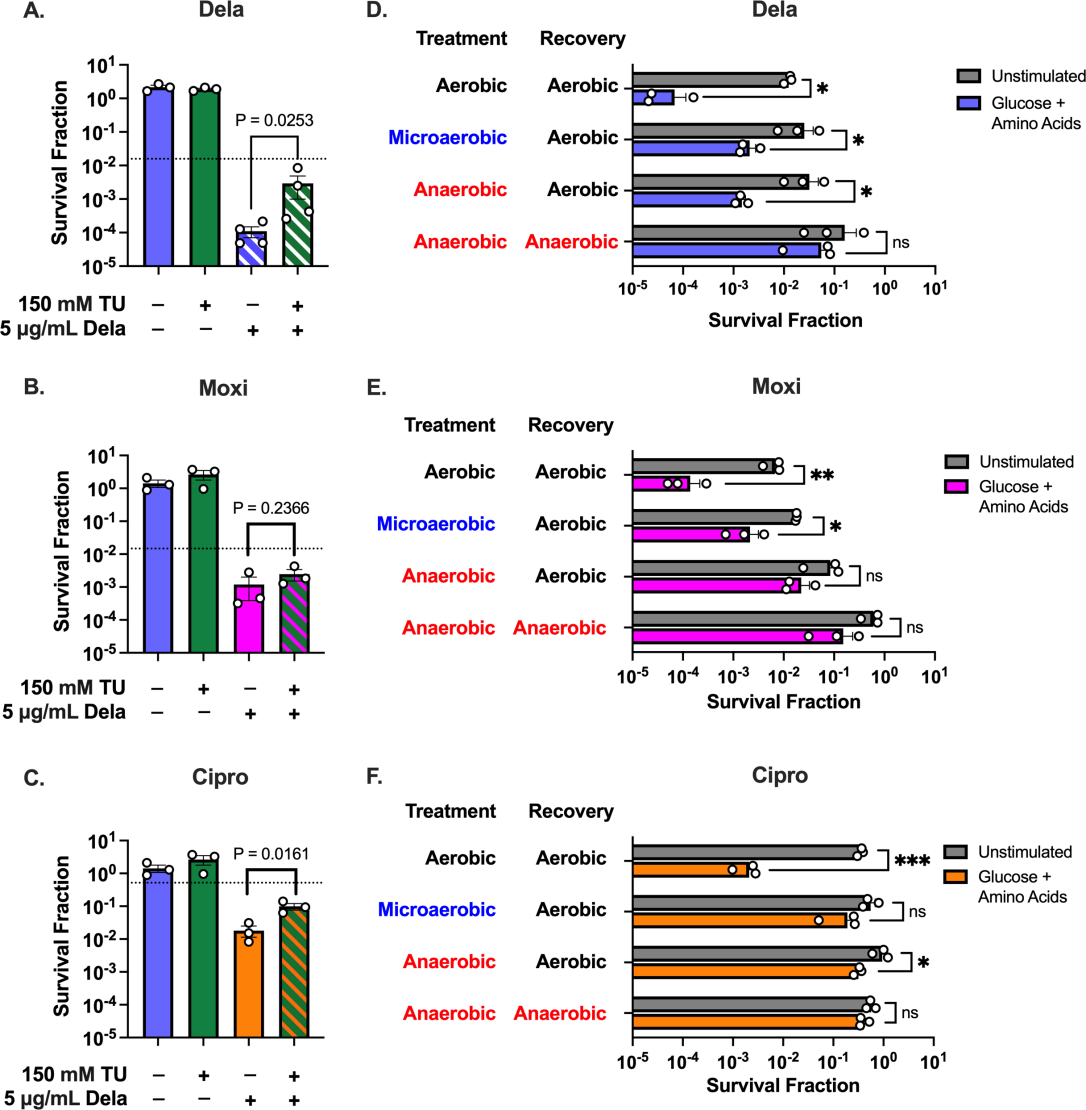
Reducing oxidative stress rescues nutrient-stimulated cells from increased killing by FQs. (**A–C**) Glucose + amino acids-stimulated cells were treated with or without TU during treatment with (**A**) Dela, (**B**) Moxi, or (**C**) Cipro. (**D–F**) Unstimulated or glucose + amino acids-stimulated cells were treated with (**D**) Dela, (**E**) Moxi, or (**F**) Cipro and recovered in environments with different levels of oxygenation (aerobic: shaking cultures, microaerobic: static cultures, anaerobic: static cultures inside an anaerobic chamber). At least three independent replicates were performed for each experiment. *P*-values were calculated using two-tailed *t*-tests to compare log-transformed values for (**A)–(C**) FQ vs FQ + TU or (**D–F**) unstimulated vs stimulated conditions. ns, not significant, *P < 0.05, **P < 0.01, ***P < 0.005, ****P < 0.0001. Error bars denote SEM.

We found that treating glucose + amino acids-stimulated cells with TU during FQ treatment protected the cells from all three FQs, but to variable extents (Fig. 6A through C). Consistent with our H_2_DCFDA data suggesting the most ROS generation in Dela-treated cells and the least in Moxi-treated (Fig. 5C through H), TU had the largest rescuing effect on Dela-treated cells (~17-fold increase in survival) and the smallest effect on Moxi-treated populations (approximately twofold) (Fig. 6A through C). TU also increased survival of unstimulated Dela-treated cells ~11-fold, a smaller effect than it had on the stimulated cells (Fig. S9A). Similar to our results for TU, Bipy increased survival of stimulated Dela-treated cells approximately fivefold while only increasing survival of unstimulated cells approximately twofold (Fig. S9B and C). Together, these data suggest that reducing oxidative stress by scavenging radicals or chelating iron has a much more pronounced effect on the survival of stimulated than of unstimulated cells, consistent with our hypothesis that glucose + amino acids enable enhanced ROS production upon FQ treatment.

While TU and Bipy are commonly used to reduce oxidative stress in studies involving antibiotics, concerns have been raised over their impact on metabolic processes (76). Although we showed that neither of these antioxidants significantly affects NADH:NAD^+^ ratio (Fig. S9D), suggesting that they do not alter redox balance, we sought stronger evidence demonstrating that ROS are the primary drivers of FQ potentiation in metabolically stimulated stationary-phase *S. aureus*. Therefore, we treated unstimulated and stimulated *S. aureus* in conditions with varying levels of oxygenation to prevent ROS accumulation during and after FQ treatment. Consistent with our prior experiments, we saw over 100-fold fewer survivors in stimulated populations that were treated with any of the three FQs compared with unstimulated populations treated in an aerobic environment (shaking culture) (Fig. 6D through F). Reducing oxygenation by treating cells microaerobically (non-shaking culture) or in an anaerobic chamber followed by recovery in an aerobic incubator reduced the difference in survival between stimulated and unstimulated Dela- or Moxi-treated cells to only 10-fold. These reductions in oxygenation were enough to essentially completely rescue Cipro-treated stimulated cells to unstimulated survival levels. Strikingly, treating stimulated *S. aureus* with Dela or Moxi anaerobically and also recovering these cells in an anaerobic environment increased survival to levels that were comparable to unstimulated cells, suggesting that ROS production not only during treatment, but also during recovery, may influence persistence (77).

Reducing oxygenation protected stimulated cells when treated with the three FQs compared to their aerobically treated counterparts (Fig. S10A through C). However, reducing oxygenation did not significantly increase the survival of unstimulated cells except in the case of Moxi-treated cells in an anaerobic environment (Fig. S10D through F). These results strongly suggest that increased ability to generate ROS upon FQ treatment underlies the increased killing of stimulated cells.

### Inhibiting nucleic acid synthesis in stimulated cells does not preclude ROS accumulation

Previous work has shown that bacteriostatic antibiotics, such as Cam, protect cells from FQs and other bactericidal antibiotics, potentially by precluding the activity of a protein required for the culmination of DSB formation upon topoisomerase stalling or preventing cellular damage that leads to ROS buildup (65, 78). Given that our data suggest that ROS are responsible for nutrient-mediated sensitization to FQs, we found it surprising that none of the biosynthesis inhibitors that we tested rescued stimulated *S. aureus*, despite their ability to decrease biosynthesis to the levels detected in unstimulated populations. To harmonize these ideas, we hypothesized that while glucose + amino acids were necessary for stimulating metabolism, leading to increased ROS, suppressing biosynthetic activities did not dampen ROS generation in stimulated cells. Consistent with this hypothesis, we found that inhibiting either RNA transcription with Rif (administered at 1.25× MIC) or DNA replication with FDC to levels equal to those in unstimulated cells did not lessen ROS accumulation following FQ treatment in stimulated cells (Fig. S11A through H). Interestingly, Rif actually enhanced ROS levels in these cells. Therefore, in addition to our earlier results showing that increased nucleic acid synthesis is not required for increased killing, we conclude from these data that increased nucleic acid synthesis is also not required for enhancing oxidative stress.

Interestingly, and consistent with previous work showing that Cam prevents ROS generation during bactericidal antibiotic treatment, we found that pre-treating *S. aureus* with Cam suppressed H_2_DCFDA fluorescence in response to FQ treatment (Fig. S11I through L) (65). In spite of this, we found that Cam only rescued stimulated *S. aureus* treated with Cipro, but not those that were treated with Moxi or Dela (Fig. 3I).

### Generalizability of findings to other *S. aureus* strains

Since *S. aureus* 43300 is an FQ-susceptible, methicillin-resistant *S. aureus* (MRSA) strain, we sought to determine whether the potentiating effect of glucose + amino acids and the rescuing effect of TU extend to other *S. aureus* strains (Table S1) (79). Specifically, we tested various doses of Dela on stationary-phase *S. aureus* strains JE2 (methicillin-resistant, FQ-resistant), Newman (methicillin-susceptible, FQ -susceptible), and SH1000 (methicillin-susceptible, FQ-susceptible) (80–82). We found that adding glucose + amino acids significantly increased Dela lethality toward these strains, and TU significantly increased survival of stimulated cells in each of these strains, albeit to varying extents (Fig. S12). Taken together, our findings show that stimulating several *S. aureus* strains with glucose + amino acids increases their sensitivity to Dela and that this sensitivity can be partially overcome by reducing oxidative stress for the strains we tested.

## DISCUSSION

Since FQs are commonly used to treat *S. aureus* infections in nutrient-limited environments, where cells are more likely to be antibiotic tolerant, we sought to sensitize stationary-phase *S. aureus* to this important class of drugs. We showed that stationary-phase cultures of multiple *S. aureus* strains, including the FQ-resistant MRSA strain JE2, can be sensitized to Dela with glucose + amino acids (Fig. 1 and 6). These findings are consistent with data from Gutierrez and colleagues, which showed that the addition of glucose sensitized stationary-phase *S. aureus* to Cipro in the presence of a terminal electron acceptor (28). However, previous work had not fully addressed how these nutrients potentiate FQs in *S. aureus*.

In our previous studies where we stimulated stationary-phase *E. coli* with glucose and sensitized the populations to FQs, we found that the added metabolites increased transcription (26). We further demonstrated that inhibiting RNA polymerase rescued these stimulated *E. coli* populations. Here, we found that the added nutrients stimulated nucleic acid synthesis in *S. aureus* (Fig. 2), but inhibiting either RNA or DNA synthesis to levels of unstimulated cells throughout FQ treatment failed to reverse the potentiating effect (Fig. 3). These findings demonstrate that discoveries made using *E. coli* may not be directly applicable to *S. aureus*, as inhibiting *S. aureus* with bacteriostatic inhibitors before treating the populations with FQs did not protect the metabolically stimulated cells from the bactericidal antibiotic (26). These data suggest that for *S. aureus*, it is not necessary to increase RNA or DNA synthesis beyond levels that were detected in the unstimulated populations to sensitize the cultures to FQs.

Previous reports have demonstrated that in exponential-phase bacteria, including *S. aureus* cultures, bactericidal antibiotics increase metabolic flux through the ETC, which leads to the production of harmful ROS (61, 62, 64). We found that stimulating stationary-phase *S. aureus* with glucose + amino acids increased ΔΨ, implying enhanced ETC activity (Fig. 4). However, unlike a previous study showing that bactericidal antibiotics increase NADH-coupled electron transport in exponential-phase *S. aureus*, our results suggest that this is not the case for stationary-phase *S. aureus* because unstimulated *S. aureus* did not show significant increases in ΔΨ upon Dela treatment (62). Additionally, further increases in ΔΨ were not observed upon Dela treatment of nutrient-stimulated cells. Therefore, while glucose + amino acids significantly increased ΔΨ in stationary-phase *S. aureus*, they did so independently of the antibiotics, marking an important difference from previous studies that were focused on exponential-phase cells.

Interestingly, while we did not detect increased ETC activity upon FQ treatment, we did observe increased ROS in nutrient-stimulated *S. aureus* when they were treated with FQs. Similar increases in ROS were not detected in the unstimulated populations. Our data strongly suggest that (1) nutrient stimulation triggers increased ROS accumulation during FQ treatment and that (2) this ROS accumulation is at least partially responsible for increased FQ lethality. Indeed, both antioxidants and limiting of oxygenation strongly enhanced the survival of stimulated cells while having comparatively little effect on the survival of unstimulated cells (Fig. 6).

In our work, we found that inhibiting RNA or DNA synthesis to unstimulated levels during FQ treatment did not reduce ROS, which may explain why inhibiting these processes to starvation levels with Rif and FDC failed to rescue nutrient-stimulated cells. We further found that while Cam depleted ROS, it rescued stimulated cells from only Cipro treatment. This difference could be due to structural differences between the FQ molecules. Our data are consistent with a previous report that a C-8 methoxy substitution, which is present on Moxi but not Cipro, allows FQs to maintain bactericidal activity against exponential-phase *S. aureus* in the presence of Cam (78). While Dela features a C-8 chloride rather than methoxy, this substitution may also contribute to Dela’s ability to overcome Cam’s protective effect (83). Additionally, in our experiments, we pre-treated *S. aureus* with 100 µg/mL of Cam (Fig. 2C). At this dose, Cam still reduced the culturability of over half the population of non-FQ-treated stimulated cells (Fig. 3G through I), implying that Cam’s effect on these cells is more far reaching and harmful than simply inhibiting protein synthesis to levels detected in unstimulated cells. While these results are interesting, protein synthesis is not the primary biosynthetic pathway targeted by FQs, and further investigation into how Cam impacts metabolically stimulated *S. aureus* is beyond the scope of this work.

While we sought to use inhibitors specific to certain biosynthetic processes, the complex feedback mechanisms in bacteria make it impossible to modulate only one variable at a time. For example, inhibiting protein synthesis most likely inhibits nucleic acid synthesis at least to some extent. Likewise, treating our cells in oxygen-deprived conditions can potentially impact metabolism and biosynthesis beyond simply precluding the generation of ROS. Despite these shortcomings, our data still present an important advance toward understanding how metabolism, biosynthesis, and oxidative stress affect killing of stationary-phase *S. aureus* by FQs.

**Fig 7:**
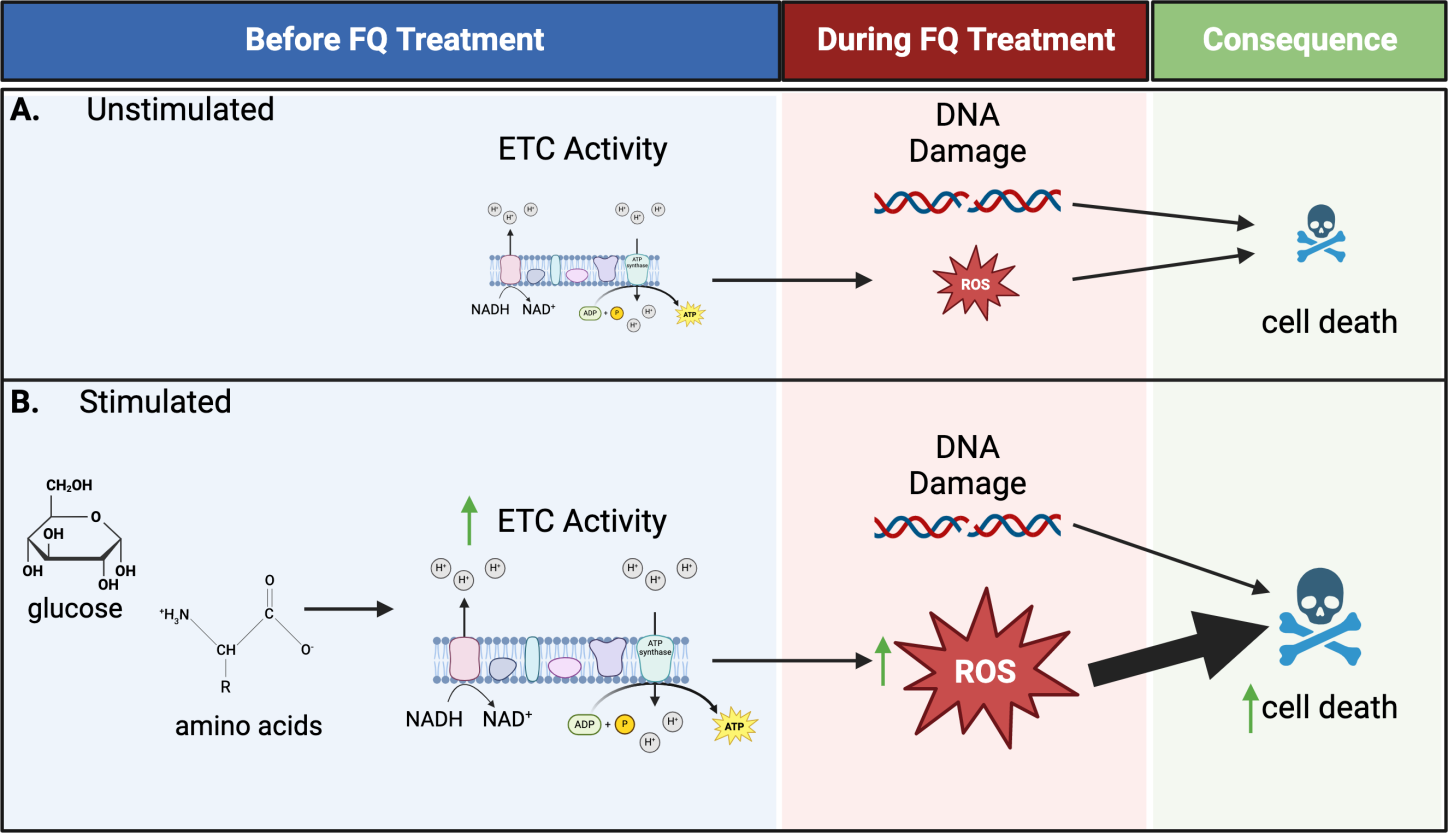
Model based on our data illustrating how nutrient stimulation sensitizes stationary-phase *S. aureus* to killing by FQs. (**A**) Unstimulated cells have low ETC activity. Therefore, while DNA damage occurs because of latent RNA/DNA synthesis, the cells are unable to generate appreciable ROS, resulting in relatively little cell death. (**B**) Upon stimulation with glucose + amino acids, cells increase ETC activity. Then, when damage occurs during treatment, the cells are primed to produce high levels of ROS, which is the main driver of increased killing. Our data suggest that nucleic acid synthesis above starvation levels is not required for increased killing of stimulated cells. Figure created with BioRender.

Based on our data, we present a model in which stimulating stationary-phase *S. aureus* with glucose + amino acids primes these cells to produce ROS upon FQ treatment, which sensitizes the cells to FQs (Fig. 7). While these nutrients, as expected, stimulate a host of biosynthetic processes, including nucleic acid synthesis, increased nucleic acid synthesis is not a requirement for enhanced killing under these conditions. Rather, we posit that the increased metabolic activity observed upon nutrient stimulation causes the cells to generate high levels of ROS during FQ treatment and that these ROS directly contribute to the increased lethality.

Our data show a correlation between increased ETC activity and increased lethality. Determining which metabolic pathways are responsible for the increased ROS generation during FQ treatment will be the subject of future studies. Since many different molecules can serve as electron donors to the ETC in *S. aureus*, it is beyond the scope of this work to test which, if any, is ultimately responsible for the increased ETC activity in the stimulated cells or whether this increased ETC activity plays a causal role in increased FQ sensitivity.

The role of ROS in controlling bacterial infections and bacterial responses to antibiotics is complex. A recent study showed that ROS generated by immune cells induce Rif tolerance in *S. aureus* by hindering *S. aureus*’s metabolism (84). We believe that these data, along with ours, suggest that exposing *S. aureus* to exogenous ROS may reprogram *S. aureus*’s metabolism and stimulate its oxidative stress responses, enabling the pathogen to better cope with bactericidal antibiotics. On the other hand, stimulating endogenous reactive metabolite production during antibiotic treatment can increase killing.

Our work on sensitizing stationary-phase *S. aureus* cultures to FQs can be used as a foundation for further research involving the nutrients required for killing of *S. aureus* by these drugs *in vivo*. Nutrient shifts at infection sites have been shown to impact *S. aureus*’s response to antibiotics. For example, a recent study showed that host inflammasome activation limits the amount of glucose available to *S. aureus*, increasing tolerance to Rif (31). Conversely, another study demonstrated that *S. aureus* degrades host collagen to peptides and free amino acids to provide energy and building blocks for growth in skin abscesses (85). Use of these liberated nutrients may influence *S. aureus*’s susceptibility to FQs in this environment by increasing the cells’ potential to generate deleterious ROS. As we gain more knowledge on the nutrient environment at different host niches, how pathogens manipulate available resources, and the impact of metabolism on antibiotic persistence, we can better predict the outcome of antimicrobial therapy and steer it toward success.

## MATERIALS AND METHODS

### Strains and growth conditions

*S. aureus* strains used in this study are listed in Table S1. *S. aureus* was grown in a chemically defined rich medium, RDM, which is modified from Teknova’s EZ Rich media and is supplemented with biotin and niacin. Additional details on growth conditions can be found in the supplemental methods.

### Antibiotic survival assays

*S. aureus* was cultured overnight in RDM and stimulated with the specified nutrients. After treatment with antibiotics, biosynthesis inhibitors, and/or antioxidants, cells were collected for colony-forming unit (CFU) enumeration. Additional details are available in the supplemental methods.

### Measuring metabolism and biosynthesis in *S. aureus*

We assessed the effects of adding glucose, amino acids, or glucose + amino acids on *S. aureus* respiration, energetics, biosynthesis, and ROS generation using established protocols, which are provided in the supplemental methods.

### Statistics

At least three biological replicates were performed for all experiments unless otherwise stated, and statistical analyses are detailed in the supplemental methods.

## SUPPLEMENTAL MATERIAL

Supplemental Material mbio.02714-24-s0001.docx Supplemental methods, figures, and tables.

ASM does not own the copyrights to Supplemental Material that may be linked to, or accessed through, an article. The authors have granted ASM a non-exclusive, world-wide license to publish the Supplemental Material files. Please contact the corresponding author directly for reuse.
