# Erratum for Batchelder et al., “Metabolites augment oxidative stress to sensitize antibiotic-tolerant *Staphylococcus aureus* to fluoroquinolones”

**DOI:** 10.1128/mbio.00073-25

**Published:** 2025-01-28

**Authors:** Jonathan I. Batchelder, Andrew J. Taylor, Wendy W. K. Mok

## ERRATUM

Volume 15, no. 12, e02714-24, 2024, https://doi.org/10.1128/mbio.02714-24. Page 12, Fig. 6B and C: “Dela” should read “Moxi” (B) and “Cipro” (C).

