# Supplementary material for: Metabolites augment oxidative stress to sensitize antibiotic-tolerant Staphylococcus aureus to fluoroquinolones
Source: mBio. 2024 Oct 30;15(12):e02714-24. doi: 10.1128/mbio.02714-24 (PMC11633220; doi:10.1128/mbio.02714-24)
Supplement: Supplemental Material — Supplemental methods, figures, and tables. [file mbio.02714-24-s0001.docx]

**SUPPLEMENTAL MATERIALS**

**Metabolites Augment Oxidative Stress to Sensitize Antibiotic-Tolerant *Staphylococcus aureus* to Fluoroquinolones**

Jonathan I. Batchelder, Andrew J. Taylor, Wendy W.K. Mok

Supplemental Methods

Tables

Table S1

Table S2

Table S3

Table S4

Figures

Figure S1

Figure S2

Figure S3

Figure S4

Figure S5

Figure S6

Figure S7

Figure S8

Figure S9

Figure S10

Figure S11

Figure S12

References

**Supplemental Methods**

*Strains and growth conditions*

*S. aureus* strains used in this study are listed in **Table S1**. Most experiments were performed with strain 43300, which is methicillin-resistant but fluoroquinolone (FQ)-susceptible (1). Strains 43300 and JE2 were subjected to whole genome sequencing to confirm their identities (SeqCenter, Pittsburgh, PA). Snippy was used to compare the genome sequences of our strains to their reference sequences (2). All *S. aureus* strains were stored in 25% glycerol at -80 °C. We inoculated strains from frozen stocks into 2 mL cation-adjusted Mueller-Hinton broth (MHBII) (Difco) in 16 x 125 mm test tubes and incubated the cultures for 3 h at 37 °C with shaking at 250 rpm. We then diluted these cultures 1:200 into 25 mL of a rich defined media (RDM), which is described below, in 250-mL baffled flasks and incubated overnight.

To ensure that the cells had reached stationary phase by 17 h under these culture conditions, *S. aureus* grown overnight in RDM was diluted to OD_600_ 0.01 in 25 mL fresh RDM and incubated at 37 °C with shaking at 250 rpm. OD_600_ measurements were taken every hour for 9 h then again at 24 h using a BioTek Synergy H1 microplate reader **(Fig. S1A)**.

For experiments in which stationary-phase *S. aureus* was stimulated with different nutrients, 17 h-old cultures were divided into separate 15-mL conical tubes and centrifuged for 10 min at 3,220 x *g*. Supernatants were decanted before the cells in each tube were resuspended in an equal volume of MOPS buffer solution containing the indicated nutrients. For experiments with exponential-phase cultures, *S. aureus* grown overnight in RDM was diluted into fresh RDM to an OD_600_ of 0.04 in test tubes and grown to OD_600_ ~0.2-0.4.

*Chemicals and culture media*

We cultured *S. aureus* in a rich, but chemically-defined, media that is based on Teknova’s MOPS EZ rich media, and we refer to it as RDM. We added glucose, niacin, and biotin to support *S. aureus* growth.

To prepare 100 mL RDM, 57 mL MilliQ water was autoclaved. 20 mL 5x EZ, 10 mL 10x MOPS, and 10 mL 10x AGCU from the Teknova kit were added to the sterilized water once it had cooled after autoclaving. Next, 1 mL 1 M D-glucose (Fisher Scientific), 1 mL 0.132 M dipotassium phosphate (Fisher Scientific), 1 mL 20 mg/mL niacin (Fisher Scientific), and 20 μL 500 μg/mL D-biotin (Fisher Scientific) were added. Final concentrations of these nutrients in RDM were 10 mM D-glucose, 1.32 mM dipotassium phosphate, 200 μg/mL niacin, and 0.1 μg/mL biotin. The combined ingredients were then sterilized using a bottle-top filter with 0.2-μm pores (Fisher Scientific).

MOPS buffer solutions containing added nutrient sources that were used to potentiate FQs were prepared so that the molarity of a given nutrient in these buffers was the same as its concentration in RDM. The concentrations of nutrients in MOPS buffer + amino acids and MOPS buffer + nucleobases are defined in **Tables S2** and **S3**, respectively. The nutrient solutions were combined with water, 10x MOPS buffer, and, when appropriate, 1 M glucose stock, then sterilized using a 0.2-μm syringe filter. Stocks of each amino acid were stored at -20 °C, and media containing glucose, amino acids, and/or nucleobases were stored at 4 °C in the dark for no longer than 1 month.

Stock solutions of antibiotics were prepared as follows: 5 mg/mL delafloxacin (Dela, MedChem Express) was dissolved in DMSO, 10 mg/mL moxifloxacin (Moxi, Fisher Scientific) in water, 16 mg/mL ciprofloxacin (Cipro, Alfa Aesar) in 0.1 N HCl, 100 mg/mL vancomycin-hydrochloride (Vanco, Fisher Scientific) in water, and 20 mg/mL 5-fluoro-2'-deoxycytidine (FDC, MilliporeSigma) in DMSO. These stocks were stored at -20 °C and were diluted to the designated concentrations before being added to cell cultures. Rifampicin (Rif, TCI) was prepared as a 20 mg/mL stock in DMSO before each use. Chloramphenicol (Cam, Acros Organics) was prepared as a 50 mg/mL stock in absolute ethanol (Fisher Scientific) and stored at 4 °C. 2-2'-bipyridine (Bipy, Sigma-Aldrich) was prepared as a 300 mM stock in absolute ethanol, and thiourea (TU, ThermoFisher) was prepared as a 1.5 M stock in water. Bipy and TU were both stored at room temperature.

*Minimum inhibitory concentration (MIC) assays*

Antibiotic test strips (Liofilchem) were used for determining the MICs of Cipro, Moxi, Vanco, and Cam for 43300 and of Dela for JE2, which is FQ-resistant (3). To measure MIC, *S. aureus* was cultured overnight in MHBII. Following overnight growth, 500 µL of cells was washed with 0.85% NaCl and diluted to OD_600_ ~0.2 in 1.5 mL 0.85% NaCl. Sterile cotton swabs were used to spread the diluted cells onto cation-adjusted MHB agar plates before the test strips were overlaid in the geometric center of each plate. Plates were incubated for 20 h at 37 °C, and the concentration on the test strip where the lawn of bacteria met the strip was considered the MIC.

Since the MICs of Rif for 43300 and of Dela for 43300, Newman, and SH1000 were below the limit of detection of the antibiotic test strips, they were determined using the broth microdilution method (4). This method was also used for determining the MIC of FDC for 43300 since no test strips are available for this drug. Overnight *S. aureus* cultures were diluted to OD_600_ ~0.01 and grown to exponential phase (OD_600_ ~0.2-0.4). Exponential-phase cells were diluted to OD_600_ 0.0001 (~5x10^5^ cells/mL) in 5 mL cation-adjusted MHB. Then, 100 μL of the culture was transferred to wells of a flat-bottom 96-well plate containing 100 µL antibiotics diluted in MHBII. Drug-free wells were included as negative controls. Plates were sealed with Breathe-Easy (MilliporeSigma) membranes and incubated at 37 °C without shaking for 20 h. OD_600_ of each well was measured, and the lowest drug concentration that inhibited growth by ~90% was determined to be the MIC. MIC values of each drug for each strain are listed in **Table S4.**

*Antibiotic survival assays*

*S. aureus* was cultured overnight in RDM as described above. Following overnight growth, cells were pelleted by centrifugation before being resuspended in equal volumes of MOPS buffer containing designated nutrients then treated with antibiotics. After a given length of antibiotic treatment, cells were collected for colony-forming unit (CFU) enumeration. Cells were incubated in test tubes at 37 °C with shaking at 250 rpm during antibiotic treatment. At the beginning and end of treatment, 100 μL culture from each tube was removed into a microcentrifuge tube containing 900 μL phosphate-buffered saline (PBS; Fisher Scientific) and centrifuged at 21,000 x *g* for 3 min. After centrifugation, 900 μL supernatant was removed, and 900 μL PBS was used to wash and resuspend the cells. This step was repeated three times to dilute the antibiotics to subinhibitory concentrations. After the final wash and resuspension, 10 μL of cells from each condition was transferred to a well in a round-bottom 96-well plate (Corning) containing 90 μL PBS, and 10-fold serial dilutions in PBS were performed. 10 μL of each dilution was plated onto Mueller-Hinton agar. Plates were incubated for 16 h at 37 °C before the number of CFUs per mL was determined. Survival fraction was calculated by dividing the CFU/mL after a given time of treatment by the CFU/mL at the beginning of treatment.

To inhibit RNA, DNA, or protein synthesis, 0.01 or 0.1 μg/mL Rif was used to inhibit transcription, 1 μg/mL FDC was used to inhibit DNA synthesis, and 100 μg/mL Cam was used to inhibit protein synthesis. Following overnight growth, *S. aureus* was resuspended in MOPS buffer or buffer with added nutrients as described above and 2 mL samples were aliquoted into test tubes. Then, the cultures were treated for 30 min with a given inhibitor or negative control (the solvent of that inhibitor). After this pre-treatment, 100 μL culture was removed from each condition to be washed and plated to calculate CFU/mL. Dela, Moxi, or Cipro was added to the remaining cells, which were treated for 7 h before FQs and/or inhibitors were diluted and CFU/mL was enumerated. *S. aureus* was treated with FQs for 7 h because we determined that this treatment duration was sufficient to kill the non-persisters in the Dela-treated populations in our time-dependent assays **(Fig. 1B).**

To determine whether ROS contribute to the increased killing caused by glucose + amino acids, 150 mM TU or 0.3 mM Bipy was added to cultures resuspended in MOPS buffer or MOPS buffer + glucose + amino acids at the time of FQ treatment. CFU/mL at the beginning and end of treatment was determined as described above.

For experiments testing the effects of aeration on *S. aureus* survival, cultures were grown overnight in RDM as described above. Then, after centrifugation, the overnight cultures were resuspended in equal volumes of MOPS buffer solutions as described above. For aerated samples, the tubes were incubated in a shaker at 37 °C, shaking at 250 rpm. For the “microaerobic” samples, the test tubes were incubated in a static incubator set at 37 °C for the duration of treatment. For the anaerobic cultures, stationary-phase *S. aureus* cells were pelleted; then, the pellets were transferred into a Coy anaerobic chamber connected to a cylinder containing 5% CO_2_, 10% H_2_, and balanced nitrogen, where they were resuspended in MOPS buffer solutions that had been allowed to equilibrate to the anaerobic atmosphere in the chamber at 37 °C overnight. All other reagents, plasticware, and pipettes that were in contact with these cultures were also deoxygenated overnight. All cultures were treated with 5 μg/mL Dela, 10 μg/mL Moxi, or 50 μg/mL Cipro for 7 h as described above. 10 µL of each resuspended culture was serially diluted and plated to determine CFU/mL at the beginning of treatment. At the end of treatment, 100 µL from each sample was transferred into microcentrifuge tube containing 900 µL PBS and centrifuged at 21,000 *x g* for 3 min. Anaerobically treated cells were kept sealed while they were being centrifuged outside the anaerobic chamber and placed back into the anaerobic chamber after centrifugation. All supernatant was removed from the centrifuged cells before the pellets were resuspended in 1 mL PBS. The cells were then serially diluted in PBS before being plated onto MHB agar. To determine whether oxygenation during post-FQ treatment recovery affects FQ lethality, we recovered anaerobically-treated cells on MHB agar both inside and outside the anaerobic chamber, allowing a comparison of survival between anaerobic and aerobic recovery, respectively, in these samples. Aerobically treated samples were diluted and recovered on MHB agar as described above. Survival fraction was calculated by dividing the CFU/mL at the end of treatment by the CFU/mL of cells from that condition at the beginning of treatment.

*Effect of nutrient sources on growth resumption*

Stationary-phase *S. aureus* stimulated with glucose + amino acids were incubated at 37 °C for 1 h with shaking at 250 rpm. OD_600_ measurements were taken every 15 min **(Fig. S2A)**, and 10 µL was removed for serial dilution to determine CFU/mL at the same timepoints **(Fig. S2B)**.

*Quantifying nucleic acid synthesis*

We used an established protocol to determine how the various nutrient sources affect nucleic acid synthesis in stationary-phase *S. aureus* (5, 6). Briefly, 2 mL of overnight *S. aureus* culture was resuspended in MOPS buffer, MOPS + glucose, MOPS + amino acids, or MOPS + glucose + amino acids in a 15-mL conical tube containing 1 μCi of ^3^H-uridine (1 μL of Revvity Stock, catalogue number NET367250UC) and incubated for 1 h at 37 °C with shaking at 250 rpm. Exponential-phase cells were used as a positive control for high RNA and DNA synthesis, and 2 mL of stationary-phase culture resuspended in MOPS + glucose + amino acids was treated with 0.01 or 0.1 μg/mL Rif to inhibit RNA synthesis or with 1 μg/mL FDC to inhibit DNA synthesis to levels comparable to those in unstimulated cultures.

For quantification of total nucleic acid synthesis, 1 mL of sample was added to 2.7 mL ice-cold 10% trichloroacetic acid (TCA) (Fisher Scientific) and incubated on ice for at least 30 min. To the other 1 mL of each culture, 110 μL of 3 M KOH (Fisher Scientific) was added to degrade alkali-labile RNA, and the sample was incubated overnight at 37 °C without shaking. Then, 2.7 mL ice-cold 10% TCA was added to these samples before incubation on ice for at least 30 min.

To collect radiolabeled nucleic acids, 25-mm diameter Whatman GF/C glass filters (Cytiva) that had been pre-soaked in 10% TCA were overlaid on a Buchner funnel connected to a vacuum tube. The TCA-precipitated samples were collected on the filters before being washed three times with 2.7 mL ice-cold 10% TCA then three times with 2.7 mL ice-cold 70% ethanol. Filters were then dried for 2 h at room temperature.

5 mL Econo-Safe scintillation fluid (Research Products International) was added to each miniature 6 mL polyethylene scintillation vial (Perkin-Elmer), and one dried filter was added to each vial. A filter with no sample was added to one vial in each experiment to account for background radioactivity. After the samples were incubated for 5 min at room temperature in the scintillation fluid, their radiation signals were measured using a Beckman-Coulter LS6500 scintillation counter and recorded as counts per minute (CPM). The CPM from the blank vial was subtracted from each sample, and the signal from the KOH-treated condition for each sample (representing radiolabeled, newly-synthesized DNA) was subtracted from the non-KOH-treated condition for each sample (representing signal from total nucleic acid) to determine the counts from newly-synthesized RNA. CPM values were then normalized to the OD_600_ of each sample measured at the onset of the experiment.

*Quantification of protein synthesis*

Protein synthesis was measured by labeling newly synthesized proteins with ^35^S-L-methionine using an established protocol with modifications (7). After overnight growth in RDM as described above, the culture was divided into 1 mL aliquots in 1.7-mL microcentrifuge tubes and centrifuged at 21,000 *x g* for 3 min. The supernatant was removed before the cells were resuspended in 1 mL MOPS buffer, MOPS buffer + glucose, MOPS buffer + amino acids (without methionine), or MOPS buffer + glucose + amino acids (without methionine). As a positive control for high protein synthesis, 1 mL exponentially growing culture in RDM was centrifuged at 21,000 *x g* for 3 min then resuspended in 1 mL pre-warmed, methionine-free RDM. To inhibit protein synthesis to levels comparable to those in unstimulated cultures, 100 μg/mL Cam was added to cells resuspended in MOPS buffer + glucose + amino acids (without methionine). To label newly synthesized protein, 500 μL of each culture was transferred to a 15-mL conical tube containing 7.5 μCi ^35^S-methionine (Revvity, catalogue number NEG709A500UC) and incubated for 1 h at 37 °C and 250 rpm.

To quench protein synthesis, 50 μL ice-cold stop solution (0.1% [wt/vol] Cam, 10 mM non-radioactive L-methionine, 0.1 M Tris-HCl [pH 7.5]) was added to each tube. Then, 2.7 mL ice-cold 10% TCA was added to each tube, and the tubes were incubated on ice for 30 min. Next, the samples were filtered through Whatman GF/C glass filter membranes that had been pre-soaked in 5% TCA using a Buchner funnel connected to a vacuum. The filter membranes containing the samples were washed three times with 2.7 mL ice-cold 5% TCA and three times with 2.7 mL ice-cold 90% EtOH before being dried for 2 h.

After drying, each filter was transferred to a scintillation vial containing 5 mL scintillation fluid as described above for measuring nucleic acid synthesis. The scintillation counter was set to read CPM from ^35^S, and background-subtracted CPM values were normalized by dividing by OD_600_ measured at the onset of the experiment.

*Adenylate nucleotide quantification*

After 17 h growth in RDM, *S. aureus* 43300 cells were centrifuged as described above and resuspended in MOPS buffer, MOPS buffer containing glucose + amino acids, or complete RDM. After 1 h incubation in these nutrients, 100 µL culture from each condition was added to 500 µL boiling propanol (Fisher Scientific) that had been preheated to 100 °C (8). At the same time, OD_600_ of each culture was measured. Cells were lysed by incubation for 5 min in propanol at 100 °C, which protects adenylate nucleotides. Then, propanol was removed using an Organomation 24-Position N-Evap Nitrogen Evaporator, and the samples were stored at -80 °C until further processing.

Concentrations of ATP, ADP, and AMP were measured using the ATP/ADP/AMP kit (Biomedical Research Service, University at Buffalo SUNY) according to the manufacturer’s instructions. Dried extracts were resuspended in 100 µL mass-spectrometry grade water. To measure total concentrations of AMP, ADP, and ATP, 5 µL of each sample was incubated with the kit’s AMP-converting enzyme and ADP-converting enzyme for 1 min to convert all AMP and ADP to ATP. 5 µL of each sample was also incubated with the kit’s ADP-converting enzyme to convert all ADP, but not AMP, to ATP. Finally, 5 µL of each sample received no converting enzyme but was incubated with water so that ATP alone could be measured. After 1 min incubation at room temperature, 35 µL ice-cold 4 mM EDTA was added to each sample. Then, 100 µL ATP assay solution was added to each sample in a 96-well Lumitrac plate, and luminescence was immediately read using a BioTek Synergy H1 microplate reader. Standard wells containing ATP solutions ranging from 0 to 50 µM were included, and luminescence readings from these standards were used to a generate a standard curve for calculating ATP concentrations in the sample wells. A blank well was included to account for background luminescence.

AMP levels in a given sample were calculated by subtracting the ATP concentration of the sample that had received only ADP-converting enzyme (ADP + ATP) from the sample that had received both ADP- and AMP-converting enzymes (AMP + ADP + ATP). ADP concentration of each sample was calculated by subtracting the ATP concentration of the sample that had been given no converting enzyme (ATP only) from the one that had been given ADP-converting enzyme (ADP + ATP). Adenylate charge was calculated using the following formula: ([ATP] + 0.5[ADP])/([AMP] + [ADP] + [ATP]) (9). Concentrations of individual nucleotides or of total nucleotides were normalized to the OD_600_ of each sample. Averages of three technical replicates were calculated for each of the three biological replicates in this experiment.

*NAD^+^ and NADH quantification*

NAD^+^ and NADH concentrations were measured as described in Schurig-Briccio et al., 2020, with modifications (10). In our experiments, we used Promega’s NAD/NADH-Glo kit. After 17 h growth in RDM, *S. aureus* was centrifuged as described above and resuspended in an equal volume of MOPS buffer containing glucose + amino acids or no added nutrients then incubated with shaking at 37 °C for 1 h.

After incubation, 500 µL culture from each condition was diluted to OD_600_ ~1 and washed twice by centrifuging for 3 min at 21,000 x *g* at 4 °C and resuspended in ice-cold PBS. After the final round of centrifugation, *S. aureus* cells were resuspended in extraction buffer made according to the kit’s directions (a 1:1 solution of PBS and 0.2 N NaOH [ThermoFisher] with 1% dodecyltrimethylammonium bromide [DTAB] [MilliporeSigma]). The cells in extraction buffer were then transferred to 2-mL Lysing Matrix Y bead tubes (MP Bio), which were shaken on a Vortex Genie 2 with a Multitube Holder attachment (Scientific Industries) at maximum speed twice for 45 s with a 2-min incubation on ice in between.

Homogenized samples were then centrifuged at 21,000 x *g* for 10 min at 4 °C. 200 µL supernatant from each tube was transferred to a new microcentrifuge tube, which was then centrifuged at 21,000 x *g* and 4 °C for 3 min to pellet residual cells and debris. The resulting supernatant was carefully transferred to another tube to ensure that beads were not carried over. The supernatants were immediately frozen using liquid nitrogen and stored at -80 °C until all samples were ready to be analyzed.

50 µL of each cell supernatant was added to two wells of a 96-well Lumitrac plate, one well for measuring NAD^+^ concentration and another for measuring NADH concentration. For NAD^+^ quantification, 25 µL 0.4 N HCl was added to each well, and the plate was heated at 60 °C for 15 min then incubated at room temperature for 10 min. Then, 25 µL 0.5 M Trizma base (MilliporeSigma) was added to each NAD^+^ well. For NADH quantification, 50 µL of a 1:1 solution of 0.4 N HCl and 0.5 M Trizma base was added to each well.

Standard wells containing 0, 5, 10, 50, 100, 200, or 400 nM NAD^+^ (MilliporeSigma) or 0, 5, 10, 50, 100, or 200 nM NADH (MilliporeSigma) were prepared by diluting these compounds in a solution containing equal volumes of PBS, 0.2 N NaOH with 1% DTAB, 0.4 N HCl, and 0.5 M Trizma base. Once the sample and standard wells had been prepared, 100 µL NAD/NADH Glo detection reagent was added to each well. Then, the plate was mixed by shaking briefly and incubated for 30 min at room temperature before luminescence was measured. Concentration of NAD^+^ or NADH in sample wells was determined using a standard curve, and NADH:NAD^+^ ratio for each sample was calculated.

*Quantification of membrane potential*

Membrane potential (ΔΨ) was measured using the BacLight Bacterial Membrane Potential Kit (Molecular Probes). Overnight *S. aureus* cultures were centrifuged and resuspended in MOPS buffer containing nutrients as described above. After 1 h incubation at 37 °C in the MOPS buffer solutions, the cultures were diluted to OD_600_ ~0.05 in 1 mL PBS containing 1 µL 3,3′-diethyloxacarbocyanine iodide (DiOC_2_(3)) reagent for a final concentration of 30 µM and incubated at room temperature in the dark for 30 min. As a control for decreased ΔΨ, cells incubated in MOPS buffer + glucose + amino acids were treated with 5 µM CCCP during the incubation period to dissipate the proton gradient.

A BD FACSymphony A5 SE cell analyzer was used to excite DiOC_2_(3) with a 488 nm laser and measure fluorescence emission intensities using 537/32 nm (green) and 602/40 nm (red) band-pass filters. DiOC_2_(3) accumulates in cells with polarized membranes and exhibits a shift from green to red fluorescence as it accumulates. Cells outside a gate set to include 99% of cells in the unstained condition were considered stained by DiOC_2_(3). The ratio of the red fluorescence intensity to green fluorescence intensity of stained cells was calculated using FlowJo.

*Quantification of ROS accumulation*

Levels of ROS were measured using the dye carboxy-2',7'-dichlorodihydrofluorescein diacetate (carboxy-H_2_DCFDA; ThermoFisher). After 1 h incubation in MOPS buffer containing the various nutrients with or without treatment with FQs and inhibitors (e.g., Rif, FDC, or Cam), cells from each condition were diluted to OD_600_ ~0.05 in 1 mL PBS containing 10 µM carboxy-H_2_DCFDA (11). These cells were then incubated for 30 min in the dark at 37 °C.

Following incubation, the cells were analyzed on a BD FACSymphony A5 SE cell analyzer, which excited the fluorophore with a 488 nm laser and measured fluorescence emission intensity using a 537/32 nm band-pass filter. As a positive control for high ROS, some cells resuspended in MOPS buffer with glucose and amino acids were treated for 1 h with 20 mM tert-butyl hydroperoxide (TBHP, ThermoFisher). To quantify the number of FQ-treated cells in each nutrient condition with ROS levels greater than those of non-treated cells given the same nutrients, we set a gate to capture 99% of untreated cells for each nutrient condition. The percentage of cells with higher fluorescence than the upper limits of these gates in the FQ-treated conditions was then calculated.

*Statistics*

At least three biological replicates were performed for all experiments unless otherwise stated. Statistical analyses were performed using GraphPad Prism version 10.1.0 and Microsoft Excel version 16.78. P < 0.05 was considered statistically significant for all experiments. For survival experiments in which only two conditions were compared, F-tests were used to confirm that variances did not differ significantly between the log_10_-transformed survival fractions of each condition. F-test comparisons in which P > 0.05 were concluded to have equal variances. Then, two-tailed t-tests assuming equal variances were used to compare the log_10_-transformed survival fractions of each condition.

For survival experiments in which more than two conditions were compared, Dunnett’s multiple comparisons test following ANOVA was used to compare the log_10_-transformed survival fractions of each condition to the unstimulated (MOPS buffer) condition or each condition to each of the other experimental conditions, as stated in the figure captions.

For all other experiments comparing only two conditions, non-transformed values were compared using two-tailed t-tests assuming equal variances (unless otherwise stated) after confirming equal variances using F-tests. For all other experiments comparing more than two conditions, Dunnett’s multiple comparisons test following ANOVA was used to compare non-transformed values as indicated in the figure legends.

**Table S1.** S. aureus *strains* *used in this study*.

**Table S2.** *Composition of MOPS Buffer + Amino Acids Solution.*

**

**Table S3.** *Composition of MOPS Buffer + Nucleobases Solution.*

**

**Table S4.** *Minimum inhibitory concentrations (MICs) of antibiotics used in this study against* S. aureus *strains.* The full range of the data is shown (n=3).

**
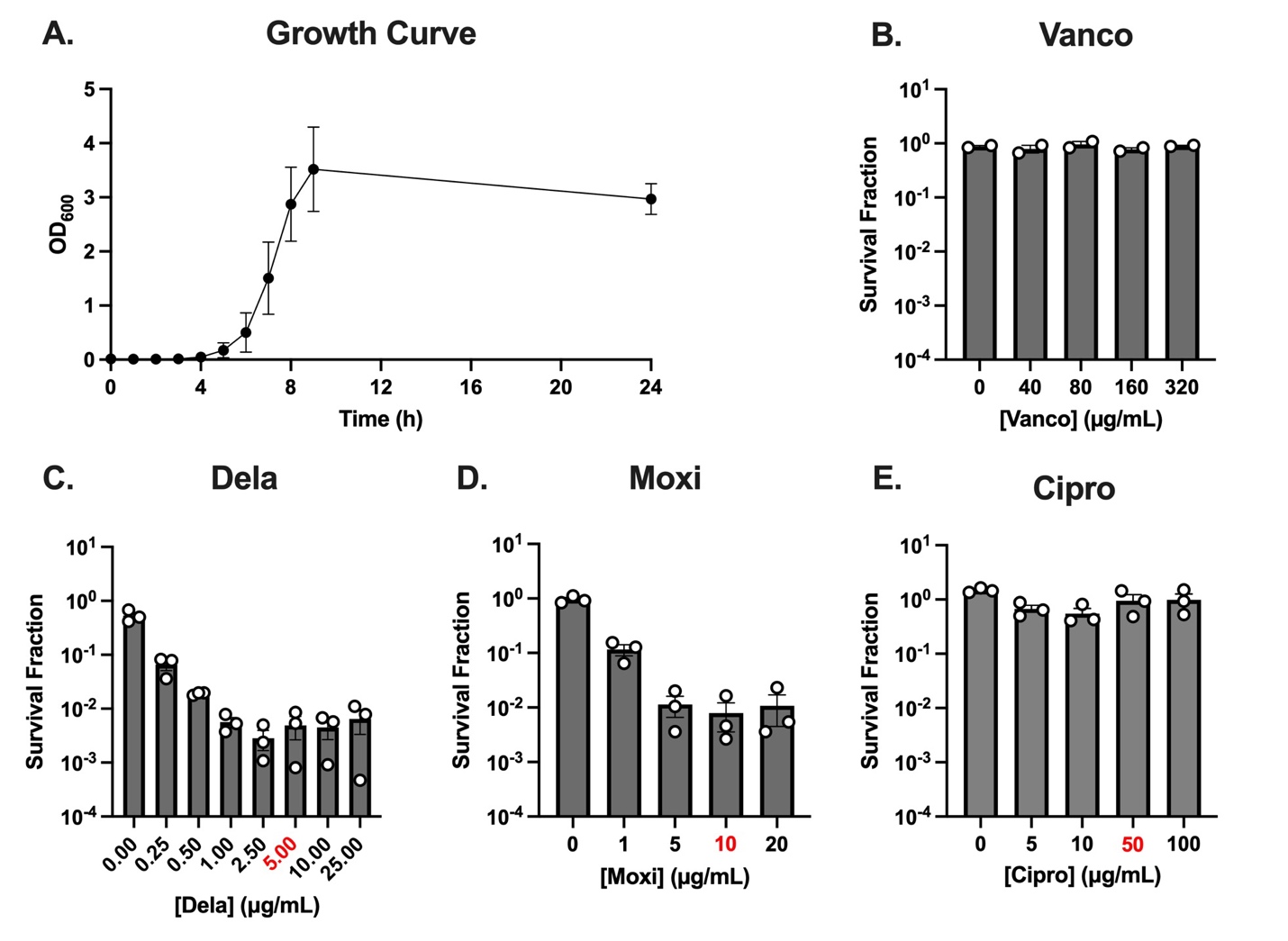
**

**Figure S1:** *Growth of* S. aureus *43300 and dose-dependent killing by antibiotics in RDM.* **(A)** OD_600_ of *S. aureus* 43300 peaks by 9 h of growth, indicating that the cells are in stationary phase by the time they are exposed to MOPS buffer containing various nutrients (after 17 h of growth) (n=3). **(B-E)** Stationary-phase (17-h) 43300 cultures in RDM were treated for 7 h with various doses of antibiotics to determine which doses should be used for further experiments. **(B)** We chose 100 μg/mL for Vanco. **(C-D)** We chose **(C)** 5 μg/mL Dela and **(D)** 10 μg/mL Moxi since these were in the second phase (plateau) of the kill curves. **(E)** We chose 50 μg/mL Cipro, which did not kill stationary-phase *S. aureus* 43300. (n ≥ 2, error bars denote SEM).


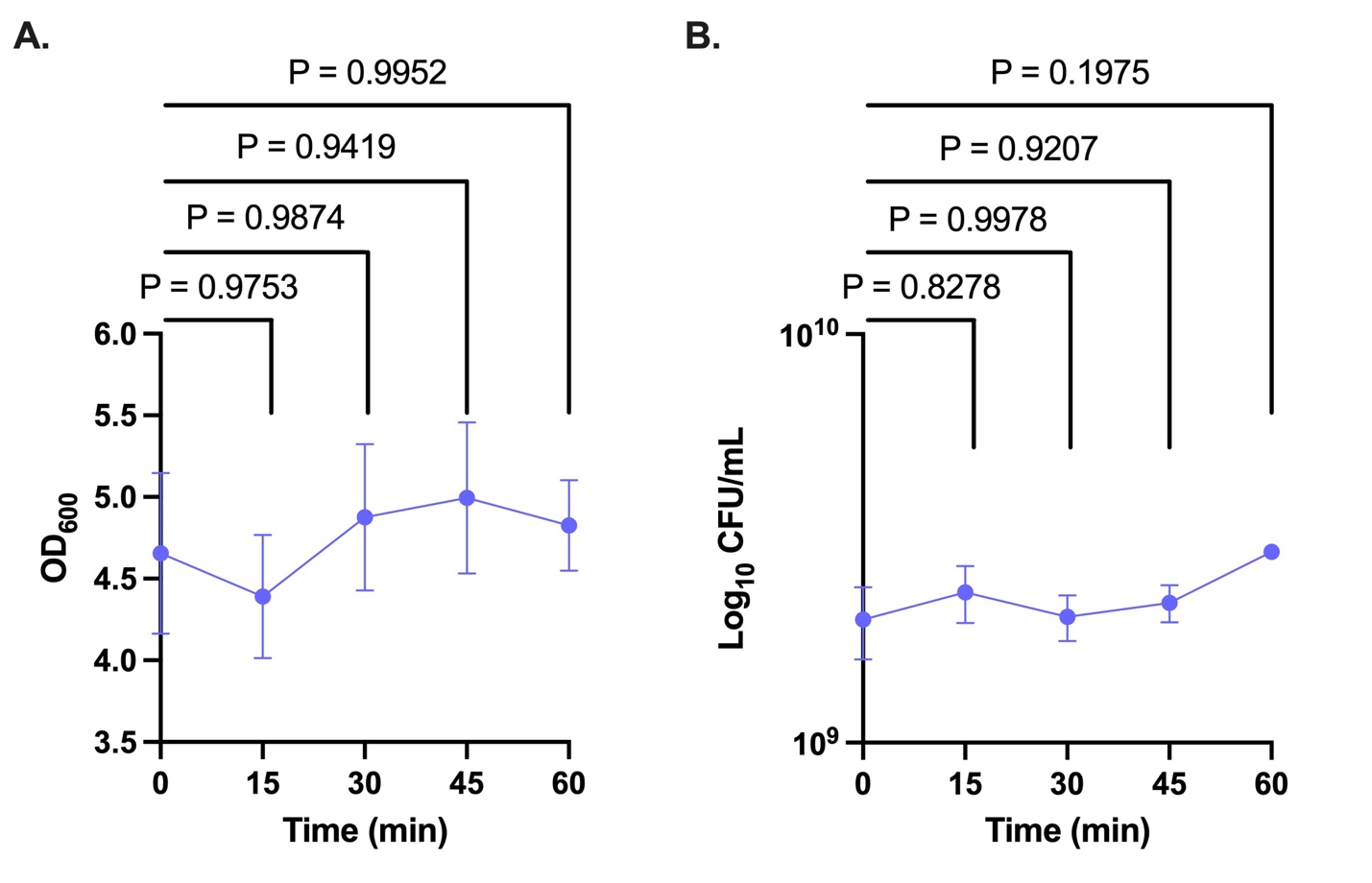


**Figure S2.** *Glucose + amino acids do not stimulate growth resumption by the time of FQ treatment.* **(A)** OD_600_ and **(B)** CFU/mL measurements were taken every 15 min for 1 h after resuspension of 17-h *S. aureus* 43300 culture in MOPS buffer containing glucose + amino acids. P values were calculated using Dunnett’s multiple comparisons test following ANOVA to compare **(A)** OD_600_ of each later timepoint to measurements taken at t = 0 (n=6) or **(B)** the log-transformed CFU/mL values of each later timepoint to the counts at t = 0 (n=3). Error bars denote SEM.


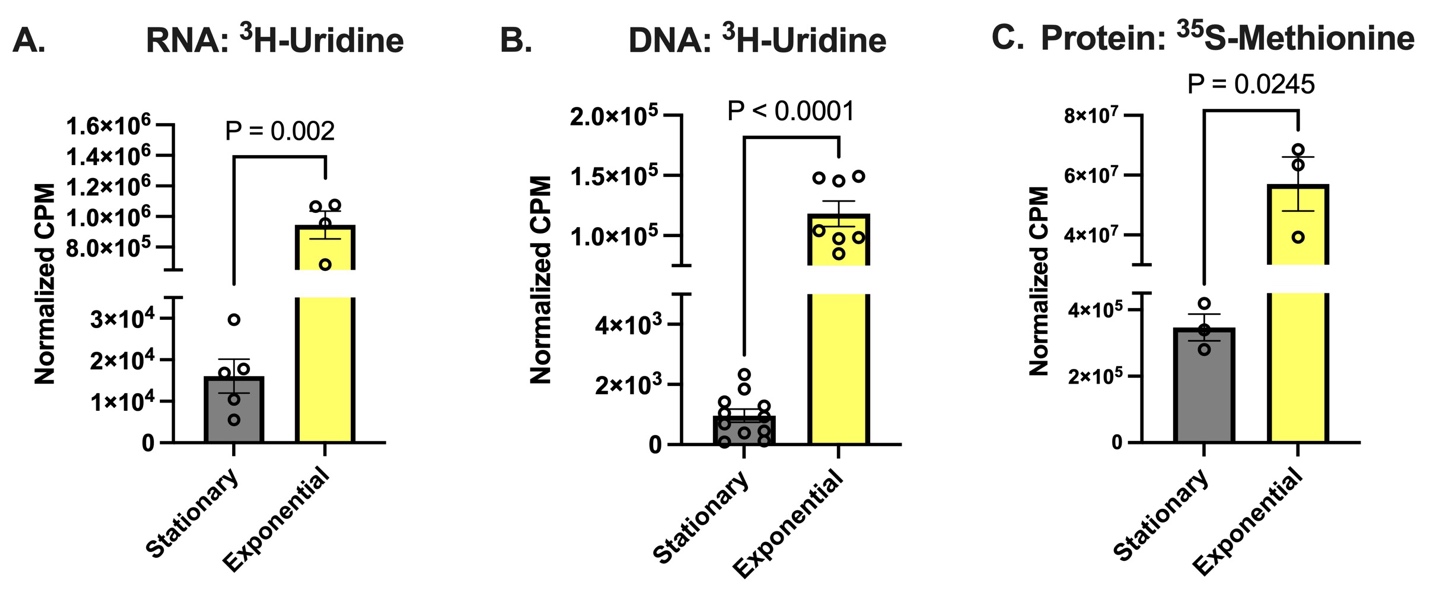


**Figure S3.** *Comparison of RNA, DNA, and protein synthesis levels between unstimulated stationary-phase and exponential-phase* S. aureus *43300.* **(A)** RNA synthesis and **(B)** DNA synthesis were measured using the incorporation of ^3^H-uridine. **(C)** Protein synthesis was measured using the incorporation of ^35^S-methionine. Radiolabeled nucleic acids and proteins were detected by scintillation counting and reported as counts per minute (CPM) normalized by the OD_600_ of each sample. At least three independent replicates were performed for each experiment. P values were calculated using t-tests assuming unequal variances. Error bars denote SEM.


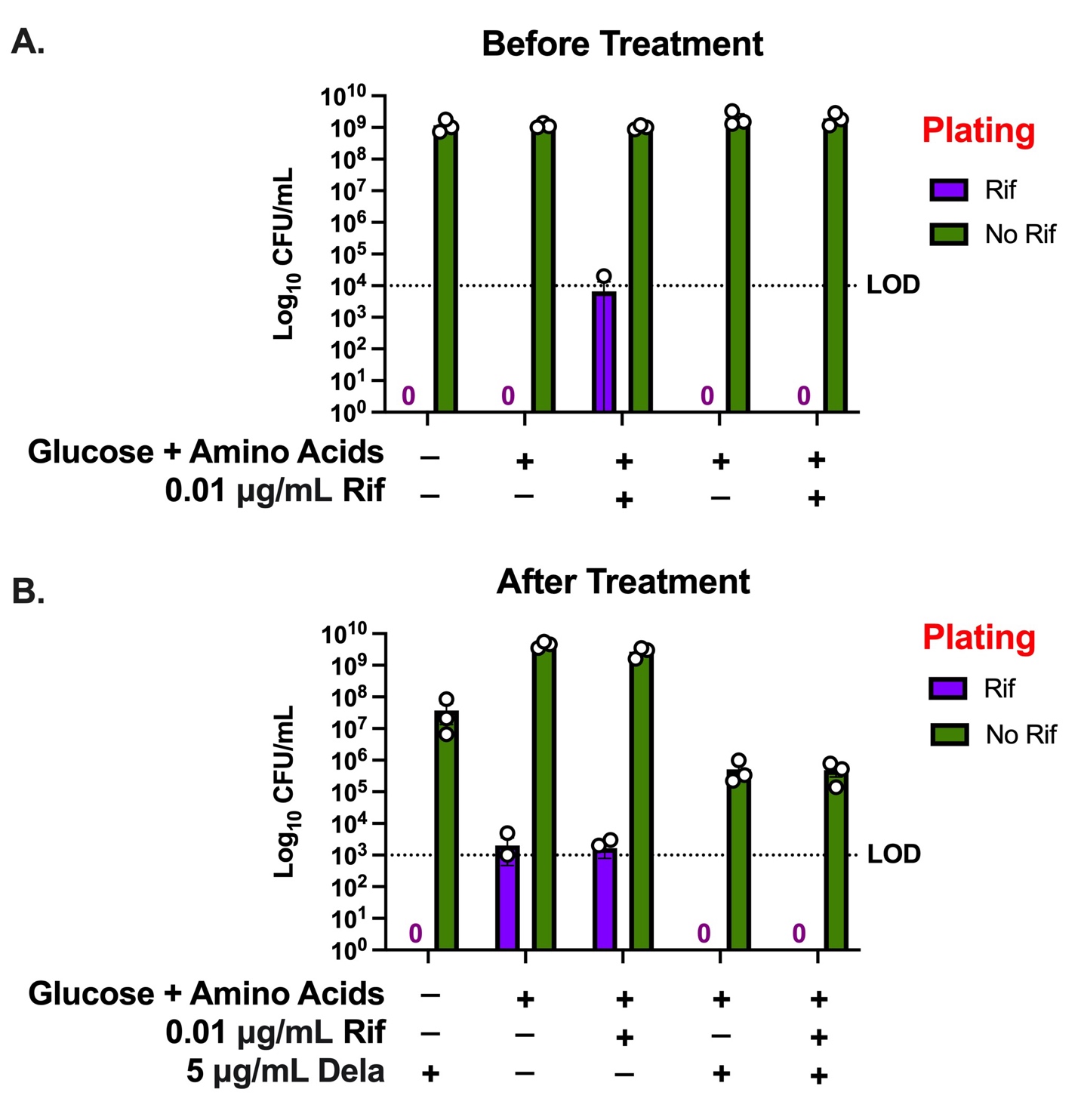


**Figure S4.** *Expansion of Rif-resistant cells is not detected in* S. aureus *43300 populations inhibited with Rif.* Unstimulated and glucose + amino acids-stimulated *S. aureus* 43300 were plated on media with or without 0.01 μg/mL Rif (the same dose used for inhibition during the rescue experiments in Fig. 2A-C) **(A)** before Dela treatment or **(B)** after Dela treatment. The number of colonies that formed on the plate containing Rif was near or below the limit of detection (LOD; dotted line) for each condition, indicating that Rif-resistant mutants make up a negligible fraction of the population both before and after treatment. Although three biological replicates were performed for each experiment, only conditions that had countable colonies for a given replicate are plotted. Error bars denote SEM.

**
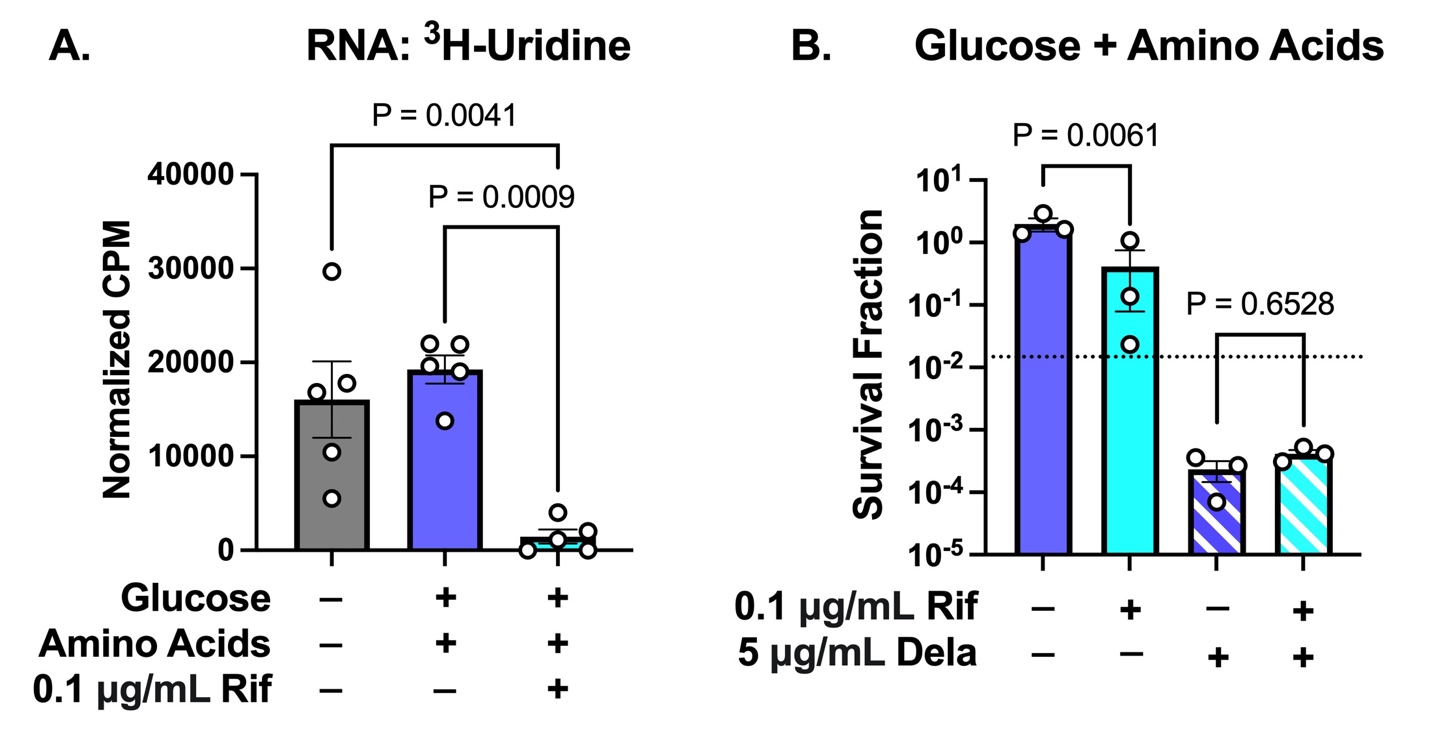
**

**Figure S5.** *Inhibiting transcription below the level in unstimulated cells is bactericidal for* S. aureus *43300 and fails to rescue from killing by Dela.* **(A)** RNA synthesis was measured using the incorporation of ^3^H-uridine. **(B)** Cells were pre-treated for 30 min with Rif during exposure to glucose + amino acids and then throughout 7 h of treatment with Dela. Dotted line indicates survival of unstimulated Dela-treated cells. At least three independent replicates were performed for each experiment. P values were calculated using **(A)** Dunnett’s multiple comparisons test following ANOVA to compare each condition to every other condition or **(B)** two-tailed t-tests to compare the indicated log-transformed values. Error bars denote SEM.


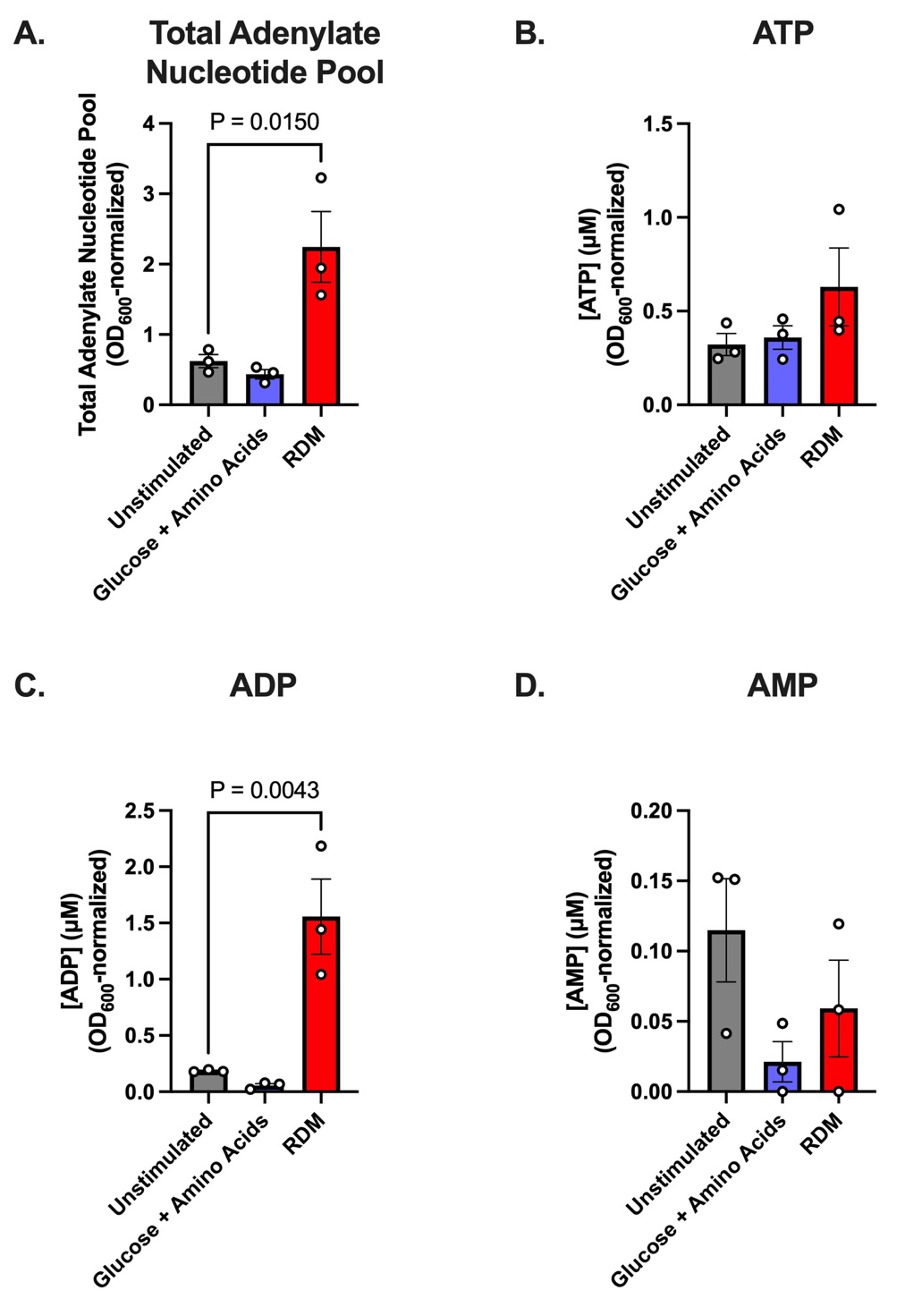


**Figure S6.** *Nutrient stimulation alters the concentrations of adenylate nucleotides in stationary-phase* S. aureus. Stationary-phase *S. aureus* cultures were incubated for 1 h with various nutrients before the quantities of **(A)** all adenylate nucleotides, **(B)** ATP, **(C)** ADP, and **(D)** AMP were measured. All concentrations were normalized to OD_600_ of their respective cultures after the hour of incubation. Three technical replicates were averaged for each biological replicate, and three biological replicates were performed for each experiment. P values were calculated comparing each experimental condition to the unstimulated condition using Dunnett’s multiple comparisons test following ANOVA. Error bars denote SEM.


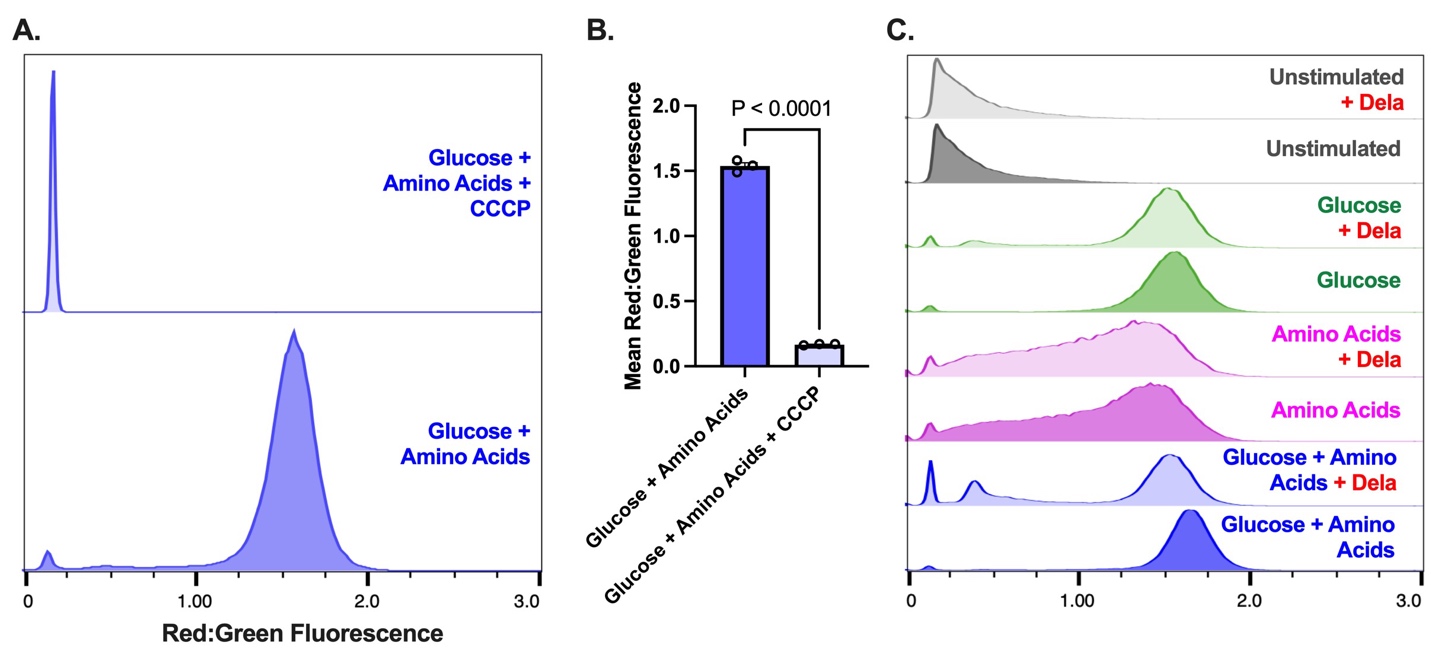


**Figure S7.** *Controls and additional data for membrane potential (ΔΨ) experiments.* DiOC_2_(3) was used to measure ΔΨ in *S. aureus* 43300. **(A)** As a control, stationary-phase *S. aureus* stimulated with glucose + amino acids was treated with CCCP, which collapsed ΔΨ. **(B)** Quantification of mean red:green fluorescence intensity of stained cells. P value was calculated using a two-tailed t-test, n = 3. **(C)** Additional replicate of the ΔΨ experiment shown in Fig. 4C-D. Histograms are representative of three independent replicates and are gated so that 99% of unstained cells are excluded.


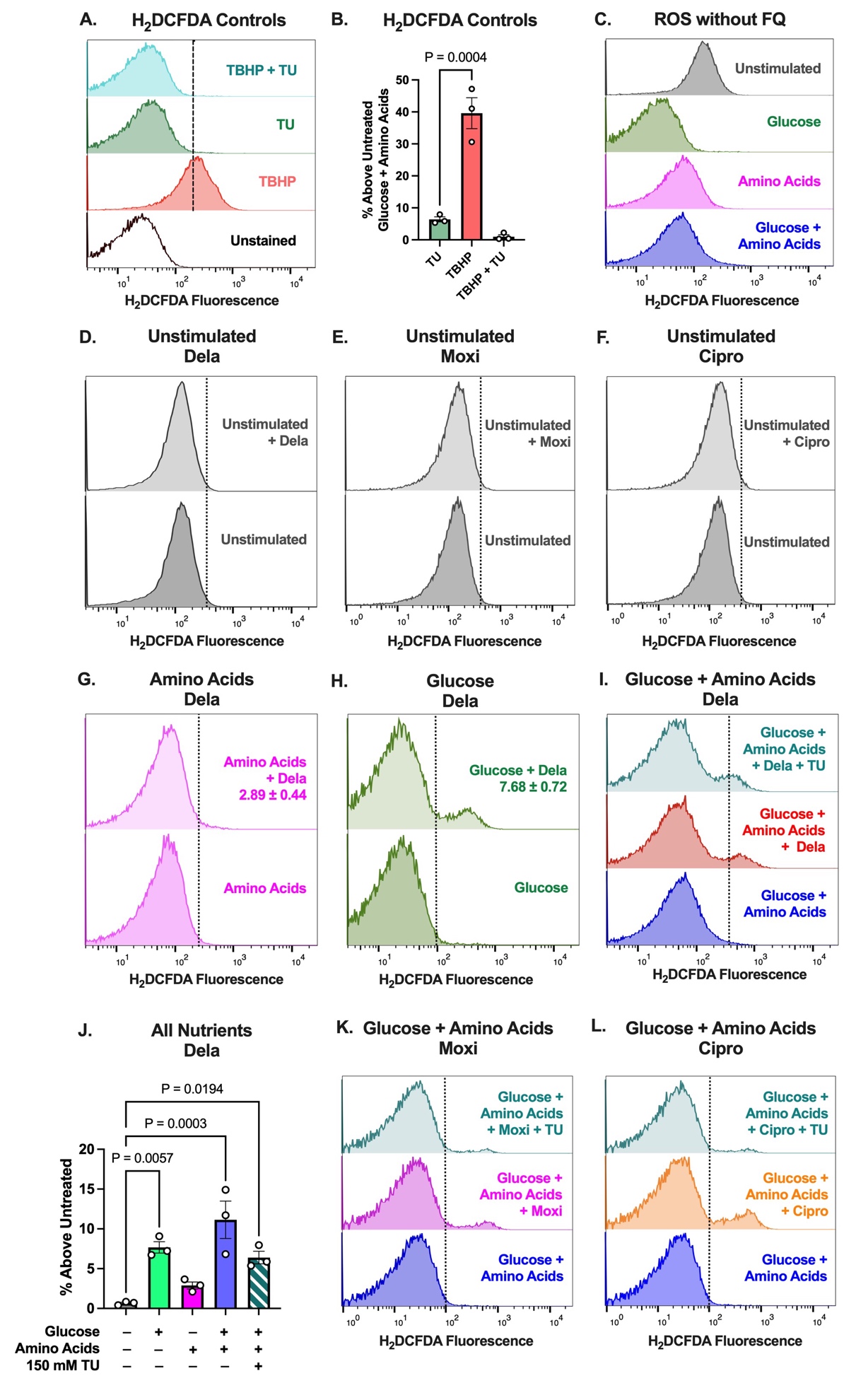


**Figure S8.** *Controls and additional data for reactive oxygen species (ROS) experiments.* Carboxy-H_2_DCFDA was used to assess the levels of ROS in *S. aureus* 43300. **(A)** Flow cytometry histogram showing unstained, unstimulated cells and stained cells stimulated with glucose + amino acids and treated with 20 mM tert-butyl hydroperoxide (TBHP), 150 mM thiourea (TU) or TBHP + TU. **(B)** Quantification of mean percentage of cells that had higher fluorescence than untreated cells. **(C)** Additional replicates of the experiment shown in Fig. 5A. **(D-F)** Flow cytometry histograms showing unstimulated cells treated with or without **(D)** Dela, **(E)** Moxi, or **(F)** Cipro. **(G-I)** Flow cytometry histograms showing cells stimulated with **(G)** amino acids, **(H)** glucose, or **(I)** glucose + amino acids and treated with or without Dela ± TU. **(J)** Quantification of mean percentage of Dela-treated cells that had higher fluorescence than non-Dela-treated cells given the same nutrients.

**(B, J)** P values were calculated using Dunnett’s multiple comparisons test following ANOVA to compare **(B)** each condition to every other condition in the panel or **(J)** each condition to the unstimulated condition (n=3). Error bars denote SEM. **(K-L)** Additional replicates of the experiment shown in **(K)** Fig. 5E and **(L)** Fig. 5G. Dashed lines indicate the gate that captures 99% of the antibiotic treatment-free cells under each nutrient condition.


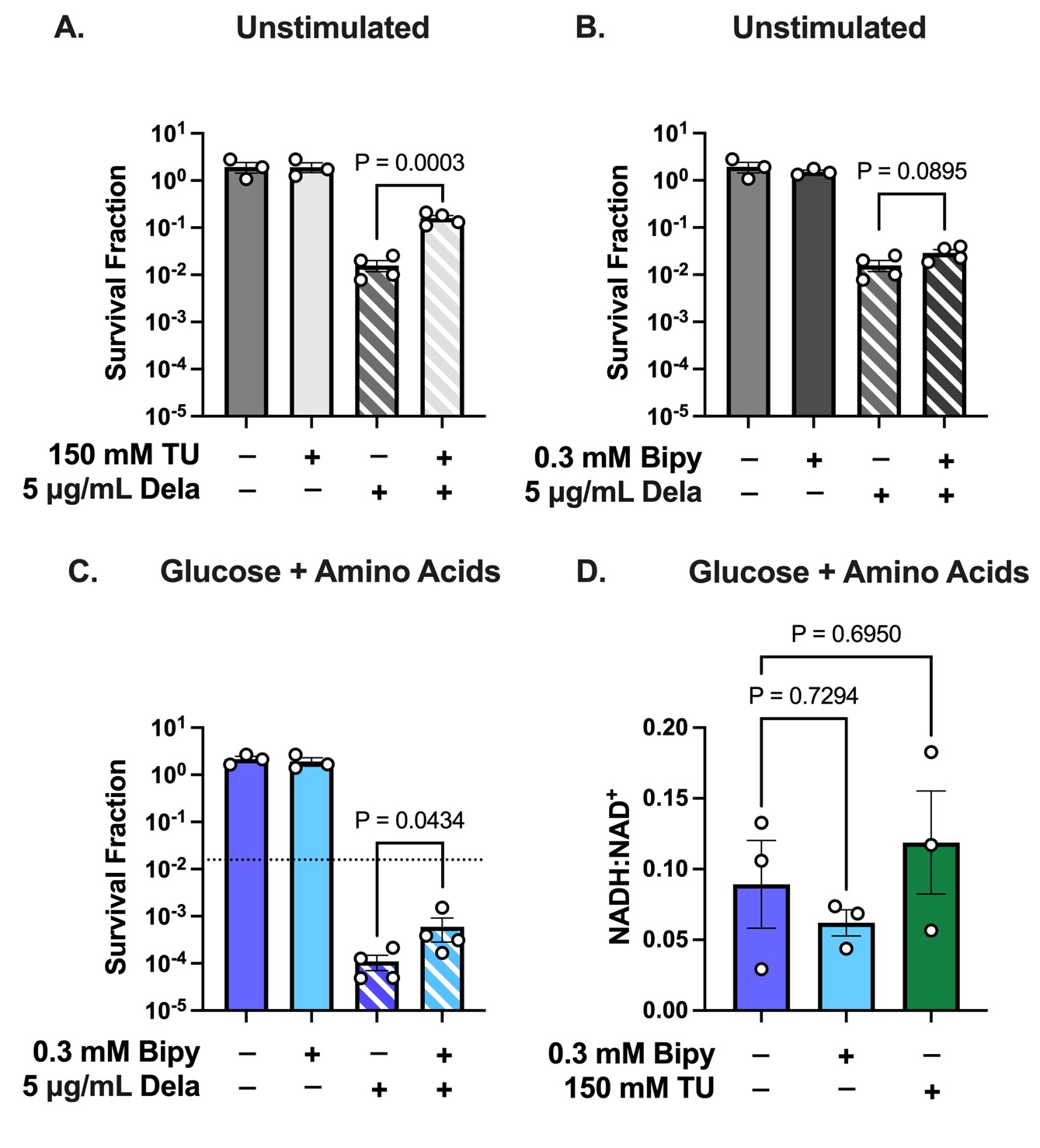


**Figure S9.** *Additional data demonstrating a role for ROS elevation in nutrient sensitization of* S. aureus *to FQs.* **(A-B)** Unstimulated cells were treated for 7 h with Dela in the presence or absence of **(A)** TU or **(B)** Bipy. **(C)** Glucose + amino acids-stimulated cells were treated with Dela in the presence or absence of Bipy. The dotted line represents the survival fraction of unstimulated cells treated with Dela. **(D)** NADH:NAD^+^ ratio of glucose + amino acids-stimulated 43300 cells in the presence of Bipy, TU, or no antioxidant. At least three independent replicates were performed for each experiment. P values were calculated **(A-C)** by comparing the log-transformed values of Dela vs. Dela + antioxidant conditions in each panel using two-tailed t-tests or **(D)** using Dunnett’s multiple comparisons test following ANOVA to compare each condition to the no-antioxidant condition. Error bars denote SEM.


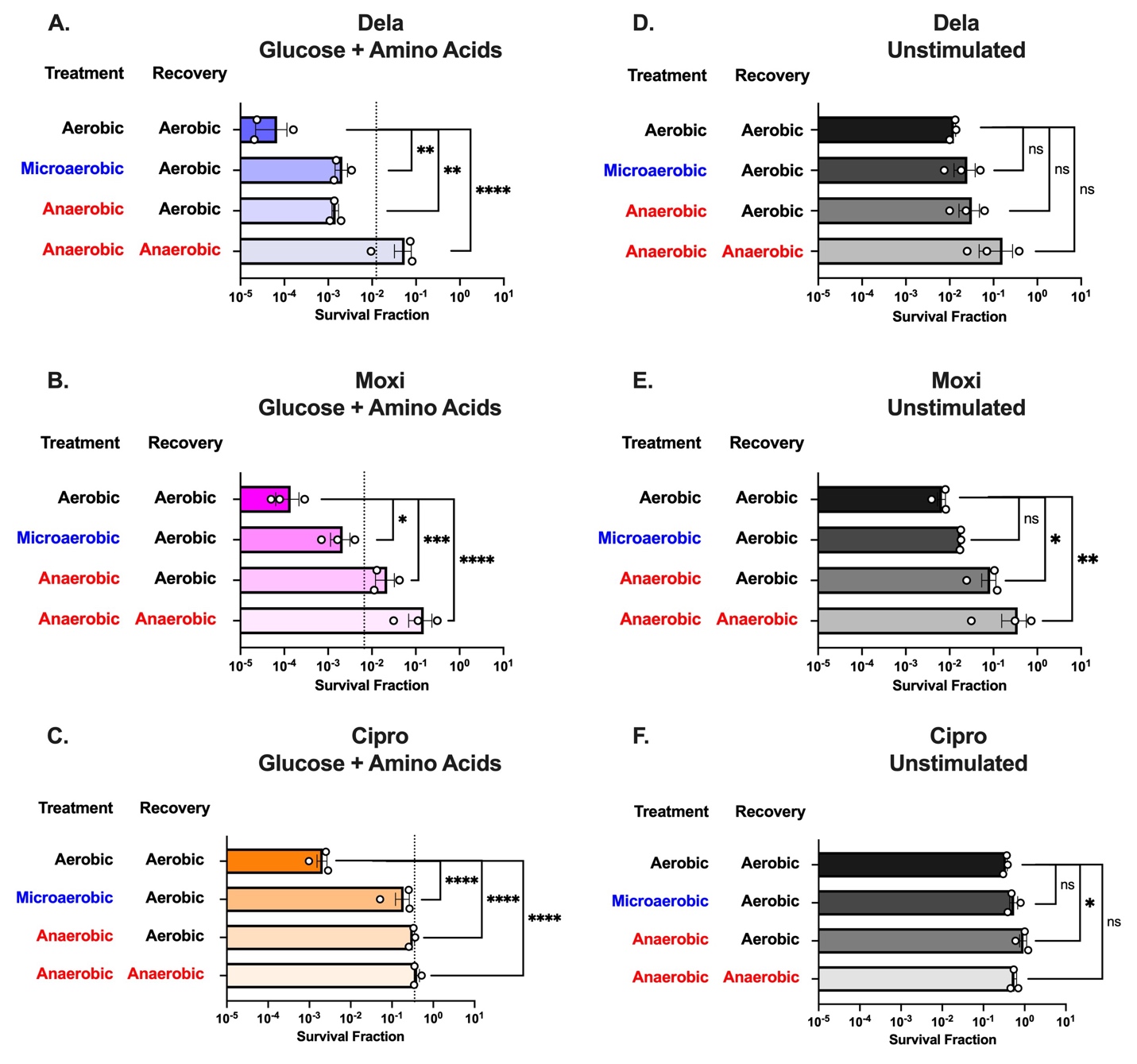


**Figure S10.** *Effect of oxygenation during treatment and recovery on the survival of stimulated and unstimulated FQ-treated* S. aureus. **(A-C)** Glucose + amino acids-stimulated or **(D-F)** unstimulated cells were treated with **(A,D)** Dela, **(B,E)** Moxi, or **(C,F)** Cipro and recovered in environments with different levels of oxygenation (Aerobic: treated in a shaken culture, recovered on an agar plate outside the anaerobic chamber; microaerobic: static culture; anaerobic: inside an anaerobic chamber). Three independent replicates were performed for each experiment. P values were calculated using Dunnett’s multiple comparisons test following ANOVA to compare the log-transformed value of each condition to the aerobic treatment and recovery condition. ns = not significant. *P < 0.05, **P<0.01, ***P < 0.005, ****P < 0.0001. Error bars denote SEM.


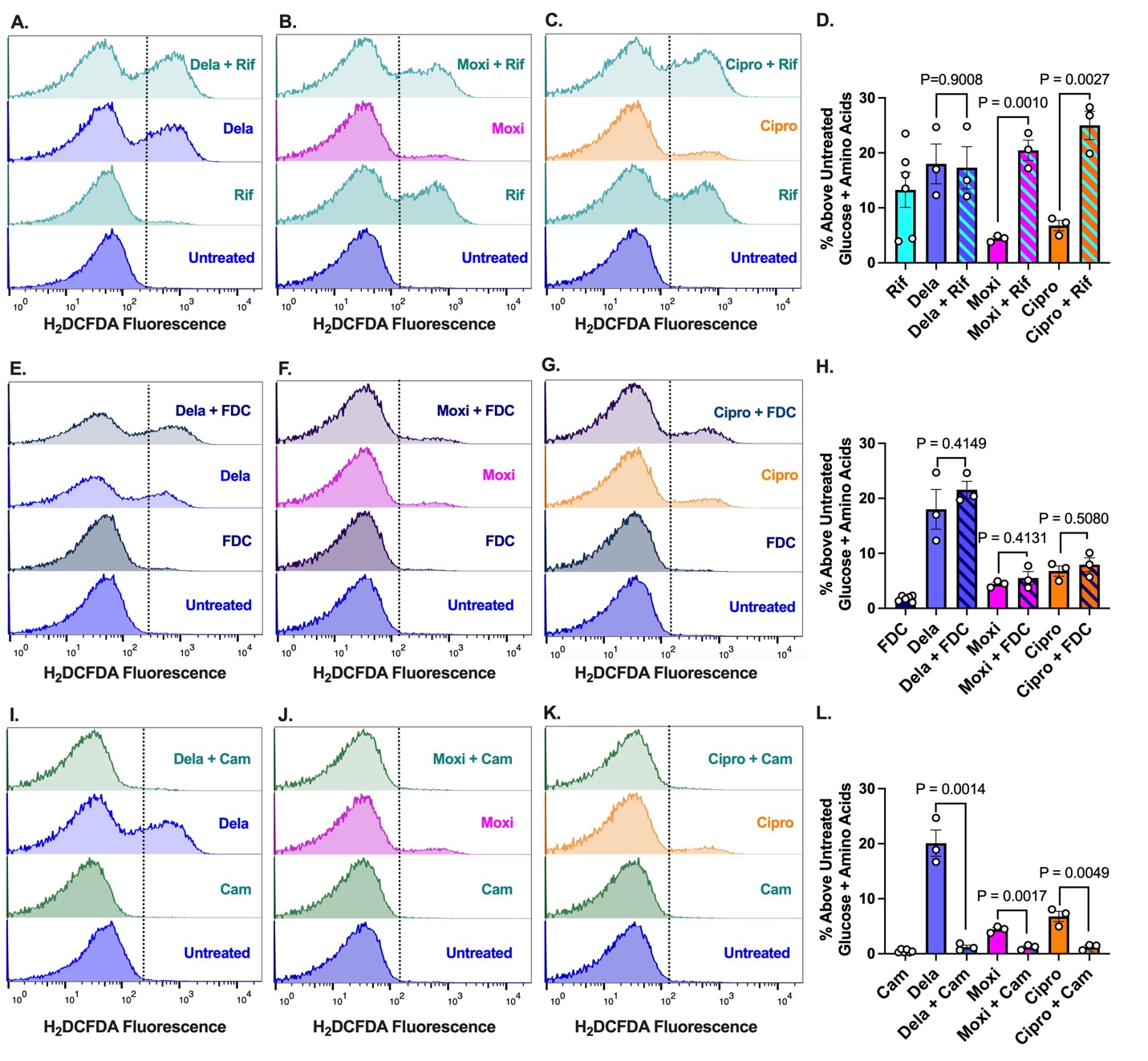


**Figure S11.** *Inhibiting nucleic acid synthesis does not limit ROS buildup in stimulated cells.* Glucose + amino acids-stimulated *S. aureus* 43300 were treated with **(A-D)** Rif, **(E-H)** FDC, or **(I-L)** Cam for 30 min before FQ treatment and throughout the subsequent 1-h FQ treatment. **(A-C, E-G, I-K)** Flow cytometry histograms (representative of three independent replicates) of H_2_DCFDA staining to assess ROS levels in stimulated cells given various biosynthesis inhibitors and FQs. Dotted lines indicate the gate that captures 99% of the antibiotic treatment-free glucose + amino acids-stimulated cells. **(D, H, L)** Quantification of the mean percentage of cells that, during treatment with a given FQ, had higher fluorescence than non-FQ-treated cells. P values were calculated using two-tailed t-tests. Error bars denote SEM.


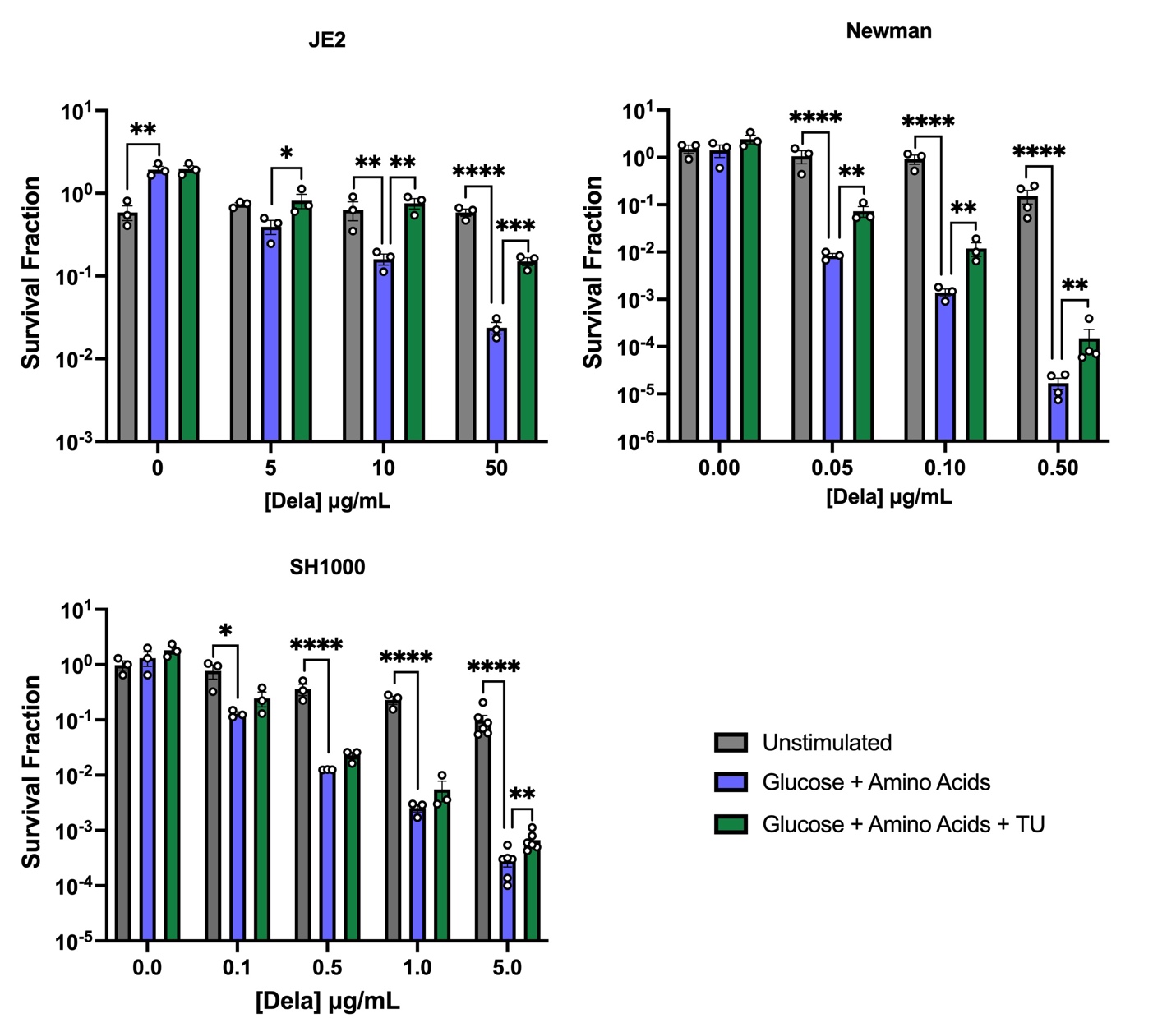


**Figure S12.** *Generalizability of nutrient-mediated sensitization to Dela in other* S. aureus *strains.* Unstimulated or glucose + amino acids-stimulated *S. aureus* strains **(A)** JE2, **(B)** Newman, and **(C)** SH1000 were treated for 7 h with varying doses of Dela with or without TU. At least three independent replicates were performed for each experiment. P values were calculated using Dunnett’s multiple comparisons test following ANOVA to compare the log-transformed value of each condition treated with a given dose of Dela to every other condition treated with that dose. *P < 0.05, **P < 0.01, ***P < 0.005, ****P < 0.0001. Only comparisons with P < 0.05 are shown. Error bars denote SEM.

**References**

1. Firsov AA, Lubenko IY, Smirnova MV, Strukova EN, Zinner SH. 2008. Enrichment of fluoroquinolone-resistant *Staphylococcus aureus*: Oscillating ciprofloxacin concentrations simulated at the upper and lower portions of the mutant selection window. *Antimicrob Agents Chemother* 52:1924–1928.

2. Seemann, T. 2016. Snippy: Rapid bacterial SNP calling and core genome alignments.

3. Vestergaard M, Leng B, Haaber J, Bojer MS, Vegge CS, Ingmer H. 2016. Genome-wide identification of antimicrobial intrinsic resistance determinants in *Staphylococcus aureus*. *Front Microbiol* 7:2018.

4. Clinical and Laboratory Standards Institute. 2021. *Performance standards for antimicrobial susceptibility testing*. 31st ed. Wayne, PA.

5. Potter AA, Musgrave DR, Loutit JS. 1982. Thymine metabolism in *Pseudomonas aeruginosa* strain 1: The presence of a salvage pathway. *Microbiology* 128:1391–1400.

6. Hare PJ, Gonzalez JR, Quelle RM, Wu YI, Mok WWK. Metabolic and transcriptional activities underlie stationary-phase *Pseudomonas aeruginosa* sensitivity to levofloxacin. *Microbiol Spectr* 12:e03567-23.

7. Reiß S, Pané-Farré J, Fuchs S, François P, Liebeke M, Schrenzel J, Lindequist U, Lalk M, Wolz C, Hecker M, Engelmann S. 2012. Global analysis of the *Staphylococcus aureus* response to mupirocin. *Antimicrob Agents Chemother* 56:787–804.

8. Setlow P, Kornberg A. 1970. Biochemical studies of bacterial sporulation and germination. *J Biol Chem* 245:3637–3644.

9. Chapman AG, Fall L, Atkinson DE. 1971. Adenylate energy charge in *Escherichia coli* during growth and starvation. *J Bacteriol* 108:1072–1086.

10. Schurig-Briccio LA, Parraga Solorzano PK, Lencina AM, Radin JN, Chen GY, Sauer J-D, Kehl-Fie TE, Gennis RB. 2020. Role of respiratory NADH oxidation in the regulation of *Staphylococcus aureus* virulence. *EMBO Rep* 21:e45832.

11. Podkowik M, Perault AI, Putzel G, Pountain A, Kim J, Dumont A, Zwack E, Ulrich RJ, Karagounis TK, Zhou C, Haag AF, Shenderovich J, Wasserman GA, Kwon J, Chen J, Richardson AR, Weiser JN, Nowosad CR, Lun DS, Parker D, Pironti A, Zhao X, Drlica K, Yanai I, Torres VJ, Shopsin B. 2023. Quorum-sensing *agr* system of *Staphylococcus aureus* primes gene expression for protection from lethal oxidative stress. *eLife* 12:RP89098

12. Keshipeddy S, Reeve SM, Anderson AC, Wright DL. 2015. Nonracemic antifolates stereoselectively recruit alternate cofactors and overcome resistance in *S. aureus*. *J Am Chem Soc* 137:8983–8990.

13. Radlinski LC, Rowe SE, Brzozowski R, Wilkinson AD, Huang R, Eswara P, Conlon BP. 2019. Chemical induction of aminoglycoside uptake overcomes antibiotic tolerance and resistance in *Staphylococcus aureus*. *Cell Chem Biol* 26:1355-1364.e4.

14. Beam JE, Wagner NJ, Lu K-Y, Parsons JB, Fowler VG, Rowe SE, Conlon BP. 2023. Inflammasome-mediated glucose limitation induces antibiotic tolerance in *Staphylococcus aureus*. *iScience* 26:107942.
